# Neural Networks of the Mouse Primary Visceromotor Cortex

**DOI:** 10.21203/rs.3.rs-4125909/v1

**Published:** 2025-04-28

**Authors:** Hong-Wei Dong, Houri Hintiryan, Muye Zhu, Pingping Zhao, Mingmin Zhang, Joshua Barry, Sumit Nanda, Mitchell Rudd, Angela Wong, Samara Miller, Lin Gou, Jinxing Wei, Brian Zingg, Jiandong Sun, Adriana Gutierrez, Hyun-Seung Mun, Ian Bowman, Luis Garcia, Darrick Lo, Tyler Boesen, Chunru Cao, Qiuying Zhao, Nicholas Foster, Keivan Moradi, Seita Yamashita, Christian Estrada, Aishwarya Dev, Jennifer Gonzalez, Hanpeng Xu, Gavin Yang, Chris Park, Xiangdong Yang, Michael Levine, Li Zhang, Paul Micevych, Carlos Cepeda, Peyman Golshani, Weizhe Hong, Yeji Yang

**Affiliations:** University of California Los Angeles; University of California Los Angeles; David Geffen School of Medicine at UCLA; David Geffen School of Medicine, UCLA; David Geffen School of Medicine at UCLA; David Geffen School of Medicine at UCLA; David Geffen School of Medicine at UCLA; University of California Los Angeles; David Geffen School of Medicine at UCLA; David Geffen School of Medicine at UCLA; USC Stevens Neuroimaging and Informatics Institute (INI), Keck School of Medicine of USC, University of Southern California, Los Angeles, CA 90033; University of Southern California; USC Stevens Neuroimaging and Informatics Institute (INI), Keck School of Medicine of USC, University of Southern California; David Geffen School of Medicine at UCLA; David Geffen School of Medicine at UCLA; David Geffen School of Medicine at UCLA; USC Stevens Neuroimaging and Informatics Institute (INI), Keck School of Medicine of USC, University of Southern California, Los Angeles, CA 90033; David Geffen School of Medicine at UCLA; David Geffen School of Medicine at UCLA; UCLA; David Geffen School of Medicine at UCLA; David Geffen School of Medicine at UCLA; Center for Integrative Connectomics, USC Stevens Neuroimaging and Informatics Institute, Keck School of Medicine of USC, University of Southern California; David Geffen School of Medicine at UCLA; David Geffen School of Medicine at UCLA; David Geffen School of Medicine at UCLA; University of California Los Angeles; David Geffen School of Medicine at UCLA; David Geffen School of Medicine at UCLA; David Geffen School of Medicine at UCLA; Center for Neurobehavioral Genetics, Jane and Terry Semel Institute for Neuroscience and Human Behavior; Department of Psychiatry and Biobehavioral Sciences; Brain Research Institute, David Geffen S; University of California Los Angeles; University of California Los Angeles; Zilkha Neurogenetic Institute, Keck School of Medicine, University of Southern California; David Geffen School of Medicine at UCLA; Jane and Terry Semel Institute for Neuroscience and Human Behavior; Department of Psychiatry and Biobehavioral Sciences; David Geffen School of Medicine at UCLA; David Geffen School of Medicine, UCLA; David Geffen School of Medicine at UCLA; David Geffen School of Medicine at UCLA

## Abstract

The medial prefrontal cortex (MPF) regulates emotions, stress responses, and goal-directed behaviors like attention and decision-making. However, the precise mechanisms underlying MPF function remain poorly understood, largely due to an incomplete characterization of its neural circuitry. Leveraging neuroanatomical, neurophysiological, and behavioral techniques, we present a detailed wiring diagram of the MPF, with a particular focus on the dorsal peduncular area (DP), an underexplored MPF area implicated in psychological stress, fear conditioning, anxiety, depression, and opioid addiction. Our analysis identifies the deep (DPd) and superficial (DPs) layers of the DP, together with the infralimbic area (ILA), as key components of the primary visceromotor cortex, that generate monosynaptic projections to regulate neuroendocrine, sympathetic, and parasympathetic functions in distinct, yet coordinated ways. Further, we demonstrate that the DP serves as a unique network hub for unidirectional cortical information flow, that integrates diverse cortical inputs and modulates social behavior. Based on the mesoscale connectome of entire MPF, we propose a unified MPF network model that regulates different aspects of motor actions associated with goal-directed behavior. This study provides novel insights into the complex role of the MPF in orchestrating physiological and behavioral responses to environmental stimuli in mammals.

## Introduction

The medial prefrontal cortex (MPF) has been extensively studied, unveiling its role in regulating emotional control, modulating autonomic and neuroendocrine responses to environmental stimuli, and coordinating stress responses ^1,2,3^. The MPF also orchestrates various facets of goal-directed behavior, encompassing temporal processes like attention, perception, motor planning, decision-making, working memory, executive function, and social interactions ^3,4,5–8,9,10,11,12^. MPF lesions are linked to a spectrum of neurological and neuropsychiatric disorders, including personality disorders, depression, post-traumatic stress disorder ^3,6,13,14,15.^ Despite these extensive studies, the precise mechanisms governing MPF functions remain unclear partly due to inconsistent circuit mapping, which creates a knowledge gap that can be bridged by a comprehensive and systematically generated network model.

Traditionally, identification of MPF regions relied on its connections to brain structures involved in autonomic and emotional responses, like the hypothalamus and periaqueductal gray ^4,16^. Accordingly, the MPF arguably includes the anterior cingulate (ACA), prelimbic (PL), and infralimbic (ILA) cortices ^8,17^. Yet, ambiguity persists in the precise definition of the rodent MPF, particularly concerning the dorsal peduncular area (DP), a unique medial prefrontal region situated between the six-layered neocortex and the three-layered olfactory cortex ^18^. Studies suggest that the DP, together with the ventrally adjacent dorsal tenia tecta (TTd), regulates autonomic and behavioral responses to psychological stress ^19,20^, anxiety and depression ^21^, fear conditioning ^22^, defensive responses ^23^, and opioid reward ^24^, which aligns with MPF function; however, a thorough understanding of its neural circuits and structural-functional position within the MPF are still needed. Without the DP, proposed unified MPF network models would be incomplete and potentially flawed.

To address this, we first redefined the anatomical borders of the DP and identified two subdivisions: the superficial (DPs) and deep (DPd) layers, each distinguished by distinct cell types and input/output connectivity patterns. Using a quantitative, comparative neuroanatomical approach, combined with electrophysiological and behavioral investigations, we constructed a comprehensive wiring diagram of the DPs and DPd, as well as other components of the MPF, including ILA, PL, and the ventral (ACAv) and dorsal (ACAd) parts of the ACA. Our findings demonstrate that the DPs, DPd, and ILA generate topographically organized projections to hypothalamic and brainstem structures to regulate neuroendocrine, autonomic, and behavioral responses important for maintaining homeostasis. Notably, this is the first report of direct cortical projections from the MPF (specifically from the DPd) to hypothalamic neuroendocrine motor neurons regulating corticotropin releasing hormone (CRH) neuroendocrine activity. Consequently, we propose that the DPd, DPs, and ILA constitute a primary visceromotor cortex. Our results also characterize the DP as a key network hub, integrating cortical inputs, mediating predominantly unidirectional cortical information flow, and displaying distinct responses to social interactions. Finally, our unified MPF structural network model lays a foundation for future investigations into MPF functions, with significant implications for understanding neurological and neuropsychiatric disorders.

## Results

### DP is a unique cortical area, juxtaposed between the neocortex and olfactory cortex with distinct anatomical and cell type features

**Figure 1:**
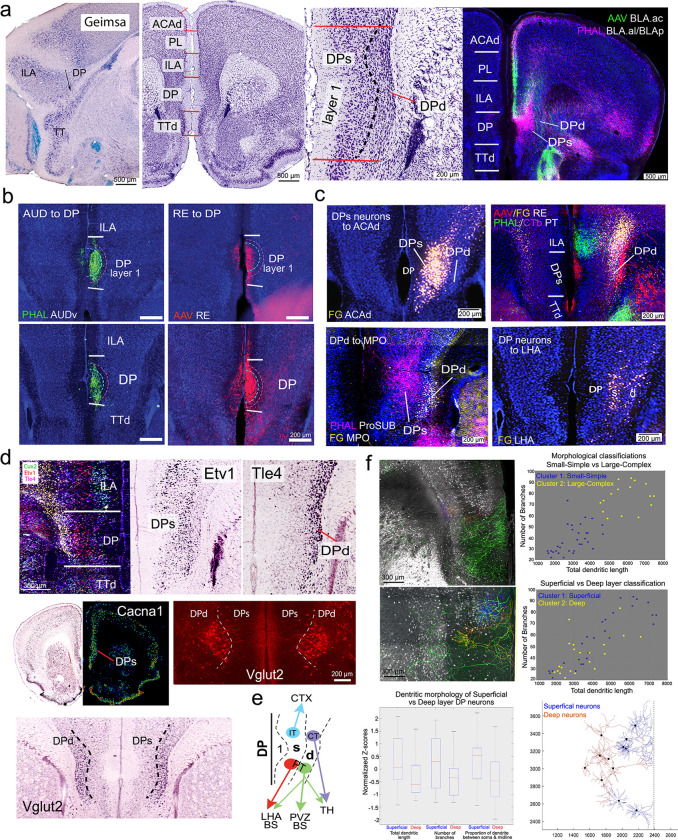
Anatomical delineation of the dorsal peduncular area (DP) and its cell type organization. **a,** DP delineation is based on combinatorial Nissl- and Giemsa-stained cytoarchitecture. DP layer 1 appears excessively thick compared to layer 1 of the ILA, PL, ACAv, ACAd, and TTd. Two primary DP sublayers are identified: the superficial layer (DPs) is characterized by relatively larger cell bodies, while the deep layer (DPd) contains densely packed smaller-sized neurons. This cytoarchitectonic feature contrasts with the TTd, which exhibits a darkly stained and densely packed layer 2 but a loosely arranged layer 3. Additionally, DP can be readily distinguished from other MPF areas by its connectivity with the amygdala. The right panel illustrates axonal inputs from the BLAal (magenta, PHAL), which generates dense terminals in the DPs. Conversely, axonal terminals from BLAam (AAV-GFP) densely innervate the ILA, PL, and DPd, but not DPs, thereby delineating the border between DP and ILA (see Extended Data Figure 2e,f for injection sites). **b,** Connectivity-based parcellation of the DP. Axonal inputs arising from AUDv and RE generate dense terminals in layer 1 of DP, defining the scope of the DP (see Extended Data Fig. 2a, c for injection sites. **c,**Connectivity-based characterization of DP sublayers. DPs, but not DPd, contains fluorogold (FG) labeled cortical neurons that project to the dorsal anterior cingulate area (ACAd, upper left). Conversely, medial preoptic area (MPO)-projecting neurons are in the DPd (lower left). Thalamic projection neurons are exclusively found in DPd (upper right), while neurons projecting to the lateral hypothalamic area (LHA) are distributed in DPd and the deep portion of DPs (lower right). Aligning with these laminar specificities, axonal projections from ProSUB are densely distributed in DPs but markedly reduced in DPd, creating a visible gap (lower left). Notably, thalamo-MPF axonal projections also exhibit regional and laminar specificity (upper right). Collectively, these connectivity data confirm the regional and laminar delineation of DP (see Extended Data Fig. 2h, j-l for injection sites). **d,**Regional and laminar specificities of DP are further validated by cell type-specific gene expression (also see Extended Data Fig. 3). The upper left panel displays a representative RNAscope image showing expression of selected marker genes: Cux2 (for cortical layer 2/3 IT cells), Etv1 (for layer 5 IT), and Tle4 (for layer 6 CT). Note that the expression of Cux2 does not extend into the DP, Etv1 is primarily expressed in DPs, while Tle4 extends into DPd (also see Extended Data Fig. 3a). These expression patterns in the DP align with those observed in the Allen Brain Atlas database (www.brain-map.org, right panel) (Extended Data Fig. 3b-g). The expression of a layer 5 marker gene, Cacna1h, is also shown with its expression extending from the neocortex into DPs but not DPd. Further distinguishing DPs from DPd is the expression of VGLUT2 that is illustrated using VGLUT-Cre mice crossed with the Ai14 reporter line and through in situ hybridization. **e,** Schematic of neuron types in the DPs and DPd. IT (to cortex) and CT (to thalamus) projection neurons are distributed in the DPs or DPd, respectively. DPd also contains PT neurons projecting to the hypothalamic periventricular zone (PVZ), while PT neurons projecting to the lateral hypothalamic area (LHA) and brainstem (BS) are distributed in both DPs and DPd. **f,** Confocal images of MORF3 labeled DP neurons reveal finely detailed dendritic morphology alongside their digital reconstructions (left panel). Many neurons extend their apical dendrites into layer 1 of DP. Morphological analysis (using k-means clustering) of the dendritic arbors (also see Extended Data Fig. 5) identifies two neuron clusters: Cluster 1 (colored in blue), which included smaller and less complex dendritic arbors (average total dendritic length: 3024 ± 833 μm, average number of branches: 38 ± 12), and Cluster 2 (colored in yellow), which included larger and more complex dendritic arbors (average total dendritic length: 5906 ± 987 μm, average number of branches: 76 ± 12). Considering the distance between soma locations and the brain's midline, we further categorized neurons as either *Deep*or *Superficial*. Integration of both classification methods reveal those deep neurons more commonly associate with Cluster 1, hence containing smaller and less complex dendritic arbor neurons, while the superficial neurons are prevalent in both Clusters, and exhibit larger and more complex dendritic wiring. *Superficial* layer neurons also display a higher proportion of their overall dendritic wiring between their cell bodies and the brain midline compared to the *Deep* layer neurons. Additionally, from each neuron, the proportions of their dendritic wiring (measured as a fraction of total dendritic length) located between their cell body and the brain midline was also quantified as a representation of overall dendritic orientation. The *Superficial*neurons contained a greater proportion of dendrites oriented towards the midline compared to the *Deep* neurons (Superficial: 66 ± 17 % of total dendritic wiring, Deep: 53 ± 17 % of total dendritic wiring, one tailed Wilcoxon Signed-Rank Test, false discovery rate corrected p=0.01). Similarly in corroboration, smaller-simpler neurons of morphological Cluster 1 were deeper (average distance from midline: 472 ± 184 μm) from the midline when compared against the larger-complex neurons of morphological Cluster 2 (average distance from midline: 337 ± 148 μm) neurons (one tailed Wilcoxon Signed-Rank Test, p=0.0099).

The DP, initially included as part of the ILA ^25^, was defined in rats as part of the olfactory cortex ^26^. In the mouse brain, the anatomical borders of the DP are inconsistent across mouse brain atlases ^8^ including the Allen Reference Atlas (ARA) ^18^ and Paxinos and Franklin mouse atlas ^27^. To address this, we refined the borders of the DP (ARA levels 37–41; **Extended Data Fig. 1a**) based on its cytoarchitecture (Fig. 1a), neuronal connectivity (Fig. 1a–c; **Extended Data Fig. 2**), and gene expression (Fig. 1d; **Extended Data Fig. 3**).

Cytoarchitecturally, the DP has a notably thicker layer 1 compared with the dorsally adjacent ILA and ventrally adjacent TTd (Fig. 1a; **Extended Data Fig. 1b; Supplementary Methods**: *Calculating MPF cortical layer 1 thickness*). The DP also exhibits a unique laminar organization that distinguishes it from other six-layered neocortical areas like the ILA. Based on Nissl and Geimsa stained cytoarchitecture (Fig. 1a), we identified two primary cellular DP layers: a superficial layer (DPs) characterized by loosely arranged, relatively larger cell bodied neurons, and a deep layer (DPd) where neurons with smaller cell bodies are densely packed. This cytoarchitectonic feature is also distinct from the densely packed, darkly stained cellular layer 2 and cell-sparse layer 3 observed in the TTd (Fig. 1a).

Our connectivity-based anatomical parcellation validated the laminar-specific DP features. Axon projections from the ventral auditory cortex (AUDv), lateral entorhinal cortex (ENTl), and reuniens thalamic nucleus (RE) generate dense terminal fields specifically in DP layer 1 (Fig. 1b; **Extended Data Fig. 2a-c**). Projections originating from the piriform cortex (PIR), anterior basolateral amygdala (lateral part; BLAal ^28^) and posterior BLA (BLAp) densely distribute in DPs (Fig. 1a; **Extended Fig. 2d-e**), while the BLAa medial (BLAam) and BLAa caudal (BLAac) generate axons in the DPd and ILA (Fig. 1a; **Extended Data Fig. 2f**). The cortical amygdala posterior lateral part (COApl) generates dense axons in DP layer 1 and DPd but avoids DPs showing a clearly distinguishable DP laminar arrangement, while injection in the COA posterior medial part (COApm) generate dense projections in the DPd (**Extended Data Fig. 2g**).

#### Connectionally-defined intratelencephalic (IT), pyramidal tract (PT), and corticothalamic (CT) cells in the DP.

Utilizing multi-fluorescent retrograde tracing, we found that IT cortical projection neurons like those that project to the ACAd and anterior COA (COAa), primarily distribute in the DPs (Fig. 1c; **Extended Data Fig. 2h-i**). In contrast, CT neurons projecting to RE and paratenial thalamic nucleus, are exclusively distributed in DPd (Fig. 1c; see **Extended Data Fig. 2j for injection sites**) ^29^. Meanwhile, hypothalamic and brainstem projecting corticofugal PT neurons, display target-specific distribution patterns. Retrogradely labeled neurons that project to the medial preoptic area (MPO) are located primarily in DPd (Fig. 1c; **Extended data Fig. 2k**), whereas those projecting to the lateral hypothalamic area (LHA) distribute in both DPd and in deeper portions of DPs (Fig. 1c; **Extended Data Fig. 2l**).

#### Molecularly defined DP cell types.

We then investigated the expression patterns of over 100 genes selected from the Allen Brain Atlas (ABA) database, confirming their regional and laminar patterns in the DP. Our RNAscope experiments with select common marker genes Cux2, Etv1, and Tle4 confirmed specificities of different DP neuron types (Fig. 1d; **Extended Data Fig. 3a**). Our analysis revealed several key findings: (1) Marker genes for cortical layer 5 (L5) IT cells, Cacna1h and Plxnd1 ^30,31^, are expressed in DPs but not in DPd (Fig. 1d; **Extended Data Fig. 3b-c**), suggesting DPs neurons share similar cell type-specific molecular features of L5 IT neurons; (2) Marker genes Tle4 and Pamr1 L6 CT or L6 CT/IT neuron types, are expressed in DPd (Fig. 1d, **Extended Data Fig. 3d**); (3) Genes Etv1/Er81 and Fezf2 that are markers for L5 PT neurons (but also for L5 IT cells) ^30,31^, are expressed in both DPd and DPs (Fig. 1d, **Extended Data Fig. 3e**). Expressions of cortical L2/3 marker genes like Cux2, or TTd marker genes like Lrmp2, do not extend into DP (**Extended Data Fig. 3f**). Additional genes like Htr2c and Fxyd6 uniquely expressed in DPs and DPd lend further evidence for their distinction (**Extended Data Fig. 3g**). Notably, combining retrograde tracing experiments with RNAscope for L5 PT marker gene Fezf2 demonstrated that a large population of PT cells in the DPd expressing Fezf2 project to hypothalamic structures such as the MPN, LHA, dorsomedial hypothalamic nucleus (DMH), and hypothalamic paraventricular nucleus (PVH) (**Extended Data Fig. 4**). Finally, we identified a unique population of neurons expressing the vesicular glutamate transporter 2 (VGLUT2) in DPd, but not in DPs or other MPF areas (Fig. 1d), which was reported to project to the parabrachial nucleus (PB) and is involved in opioid reward ^24^.

These findings highlight that the DPs is characterized by the presence of L5 IT-type neurons, which project to other cortical areas and the amygdala, and PT-like neurons, which project to the LHA (Fig. 1e). On the other hand, the DPd predominantly contains CT- and PT-type neurons, with the latter primarily projecting to periventricular hypothalamic structures like the MPO, as well as the brainstem structures, such as PB.

#### Morphological DP cell types.

Single-neuron reconstruction technologies examining the morphological features of MPF neurons ^32,33^ have excluded the DP. We combined the genetic sparse labeling MORF3 ^34^ reporter line with brain clearing and 3D microscopic imaging to acquire the morphological details of sparsely labeled excitatory DP projection neurons (Fig. 1f; **Extended Data Fig. 5**), to systematically characterize their morphological features (see Methods). 48 reconstructed DP neurons were categorized into two morphological clusters based on dendritic size (quantified by dendritic length) and complexity (quantified by number of branches) (Fig. 1f; **Extended Data Fig. 5**). Cluster 1 included smaller, less complex neurons (32 neurons, average total dendritic length: 3024 ± 833 μm; average number of branches: 38 ± 12). Cluster 2 contained larger, more complex dendritic arbors (16 neurons, average total dendritic length: 5906 ± 987 μm; average number of branches: 76 ± 12). Next, DP neurons were classified into *Superficial* vs. *Deep* layer clusters based on the distances of their cell bodies from the midline. Comparing *Deep* (21 neurons, distance from midline: 605 ± 106 μm) and *Superficial* (27 neurons, distance from midline: 289 ± 79 μm) clusters (Fig. 1f; **Extended Data Fig. 5**) revealed that *Deep* neurons were smaller and less complex (average length: 3472 ± 1285 μm; average number of branches: 42 ± 16), whereas the arbors of *Superficial* neurons were larger and more complex (average length: 4383 ± 1775 μm; average number of branches: 57 ± 24). 86% of *Deep* neurons (18/21) fell within the smaller and less complex morphological class (Cluster 1). In contrast, *Superficial* DP neurons fell across both (larger-complex and smaller-simpler) morphological clusters. Overall, the *Superficial* cluster neurons were both larger (one-tailed Wilcoxon Signed-Rank Test, false discovery rate corrected p=0.04) and more complex (p=0.023) compared to *Deep* cluster neurons. These results align with the cytoarchitectonic Nissl data suggesting distinct cell type-specific morphological features of DPs and DPd neurons.

### Distinct global neural network features of the DPs and DPd distinguish them from other MPF components

Extensive research has been conducted on MPF connectivity in rats and primates ^4,35,36,37^. In mice, investigations have focused on cortical and thalamic MPF connectivity at regional, cell type-specific, and single neuron resolutions ^9,32,33,38,39,40,41^, although the global neural network of individual MPF regions, including the DP, has not been systematically generated.

Our well-established data production and annotation pipelines were implemented to investigate the brain-wide input/output connectivity of the DPd, DPs, ILA, PL, ACAv, and ACAd. Anterograde (AAV, PHAL, BDA) and retrograde (CTB, FG) tracer injections were placed into each MPF region (**Extended Data Fig. 6a-b,** see Methods and **Supplementary Material** subsection *Overview of connectivity data collection, analysis, and validation*). Image data were registered, and tracer labels were thresholded and annotated using our in-house software, *Outsepctor* ^28,42,43,44,45^ (**Extended Data Fig. 7a-e**; **Supplementary Methods** subsection, *Outspector: Our proprietary 2D image processing pipeline*). To ensure consistent labeling, 2D hierarchical clustering was performed on data from multiple injections into individual ROIs (**Extended Data Fig. 7f**). Annotated data were normalized and analyzed, with visualizations of anterograde, retrograde, and reciprocal connections presented in connectivity matrices. Aggregated connectivity data from individual cases with injections in each MPF area—DPs, DPd, ILA, PL, ACAv, and ACAd—were quantitatively analyzed to construct 2D hierarchical clustering for the Projection Fraction Matrix, Input Fraction Matrix, and Reciprocity Fraction Matrix for each MPF area (see **Supplementary Methods** subsections: *Statistical model of brain connectivity* and *Quantifying the overview of MPF whole brain connectivity;* and **Supplementary Results**). These matrices facilitate direct comparison of the global connectivity patterns of these areas, suggesting that each of them displays distinctive input/output patterns (**Extended Data Fig. 8a-b;** also see **Supplementary Results**). Global connectivity pathways of these MPF areas were also depicted on brain flatmaps **(Extended Data Fig. 9**), and an online tool is available for viewing all reconstructed connectivity maps (https://brain.neurobio.ucla.edu/mpf/ ; username: guest, password: mpfbrainmap710).

**Figure 2:**
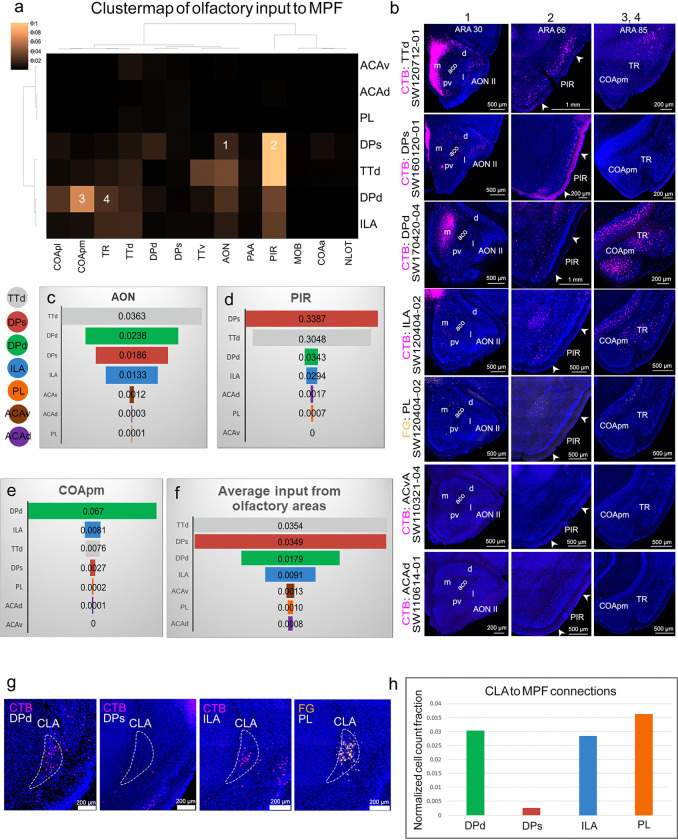
Connectional input specificities of MPF areas with olfactory cortical areas and the claustrum (CLA). **a**, A 2D cluster map highlights the distinctive olfactory inputs to MPF areas and TTd, based on the connectivity data shown in **b**(for injection sites, see Extended Data Fig. 6). The brightness of the grids corresponds to strength of input. The brighter the grid, the stronger the connection. Numbers 1–4 refer to the corresponding panels of retrograde labeling in different olfactory regions in **b**. Note the consistency between the raw image data and the results of the clustermap. For example, strongest input to MPF from the COApm (#3) is to the DPd, which is evident from both the clustermap in **a** and the raw image in **b**. **c-e**, Quantitative comparisons of olfactory inputs to different MPF areas from the AON, PIR, and COApm, respectively. **f**, A quantitative summary of average projections to MPF areas from those olfactory regions. Collectively, these data illustrate distinct connectivity patterns of the TTd, DPs, DPd, and other MPF areas. While TTd (as part of the olfactory cortical area) receives dense inputs from the PIR, AON, and TTv, DPs receives dense inputs from the PIR and AON. DPd uniquely receives dense inputs from the COApm, in addition to other olfactory inputs. ILA also receives weak olfactory inputs, while other MPF areas remain void of olfactory inputs. **g**, Retrograde labeling in the CLA after CTB or FG injections into the DPd, DPs, ILA, or PL reveals quantitatively distinct connectivity patterns shown in **h**. DPs receives no inputs from the CLA, whereas DPd, ILA, and PL receive robust inputs from the CLA. Also note the consistency between the raw images and the quantified data. **See Extended Data Fig. 11 for connectional output specificities of MPF areas with cortical areas and the CLA.**

Our project primarily utilized multi-fluorescent tract-tracing methods, including anterograde tracers (e.g., PHAL, AAV), retrograde tracers (CTB, FG), and viral tracers (genetically engineered rabies). To address tracer tropism, individual variability, and ensure the accuracy, specificity, and reliability of our connectivity data, we conducted extensive validation experiments. This rigorous approach was particularly crucial given the proximity of the DPs and DPd to each other, as well as their adjacency to two other relatively small structures, the ILA and TTd. Firstly, we analyzed cortico-thalamic connections arising from well-studied MPF areas, such as ACAv, PL, and ILA, to the midline/medial thalamic nuclei (**Extended Data Fig. 10)**. Our findings showed strong congruence with the existing literature ^36,39,40,46^. Next, we performed quantitative analysis of inputs and outputs of MPF areas and the TTd with different olfactory cortical areas, including the main olfactory bulb (MOB), anterior olfactory nucleus (AON), piriform cortex (PIR), and cortical amygdalar area (COA) (Fig. 2a–f; **Extended Data Fig. 11a-f**). These analyses revealed (1) TTd, like other olfactory regions, projects directly to the MOB and exhibits extensive reciprocal connections with other olfactory areas, such as the AON and PIR; (2) DPs and DPd receive extensive inputs from olfactory areas (e.g., AON, PIR) but generate much weaker projections back to these regions. Unlike TTd, they do not project to the MOB. Notably, DPd, but not DPs, receives dense inputs from the COA posterior medial part (COApm), which processes accessory olfactory information ^47^; (3) in contrast to DP, the ILA receives significantly fewer olfactory inputs and lacks input from the COApm. Other MPF areas exhibit minimal or no connectivity with olfactory regions. In addition, our data demonstrated distinct input/output patterns between MPF areas with the claustrum (CLA) (Fig. 2g–h; **Extended Data Fig. 11g-h**): (1) The ILA and PL maintain strong bidirectional connections with the CLA, as reported previously ^38^; (2) DPd receives inputs from the CLA but does not project back to it. But DPs show no connectivity with the CLA.

Supplementary injections were made across additional cortical areas, as well as hippocampal, amygdalar, thalamic, hypothalamic, and brainstem structures (elaborated in corresponding sections below). This strategy allowed for the detailed examination of global MPF connectivity, ensured reliability through precise validation of injection sites, and enabled cross-validation between anterograde and retrograde datasets. AAV1-Cre-based transsynaptic, Cre-dependent, and TVA-mediated rabies tracing data were also incorporated to enhance and expand the MPF multi-synaptic and cell type-specific network analysis, as detailed in the corresponding sections.

Altogether, our data suggested that the DPs and DPd display distinct global input/output connectivity patterns, which distinguish them from the TTd, ILA and other MPF areas (**Extended Data Figs. 8–9**). Refer to **Supplementary Results** for quantitative analysis of (*1) MPF connectivity with cortex;* (*2) Distinctive characteristics of DP's cortical connections, and (3) Unique cortical connections of other MPF areas*.

#### DPs and DPd receive distinct cortical inputs.

**Figure 3:**
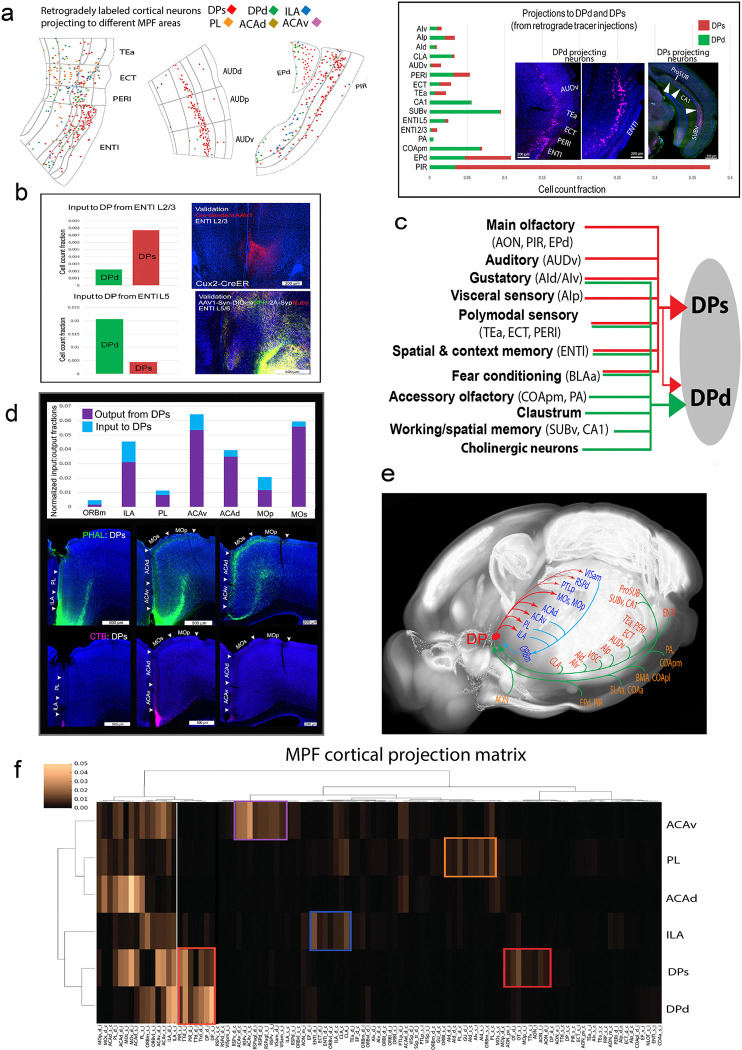
Cortical input/output organization of the DPs, DPd, and other MPF areas **a,** Left: Distribution map of cortical neurons projecting to the DPs, DPd, and other MPF areas reconstructed from retrograde tracing experiments. The map shows a broad spectrum of cortical inputs to the DPs and DPd. Right: Bar graph illustrating comparative inputs to the DPs and DPd based on retrograde tracing results. Representative micrographs of CTB labeled DPs or DPd projecting neurons are also shown. See Extended Data Figs. 12–13 for retrograde tracing data and map). All retrograde cases can also be found on the online map (https://brain.neurobio.ucla.edu/mpf/ ; username: guest, password: mpfbrainmap710). **b,** Using anterograde tract tracing method, we validated quantitatively distinct inputs from ENTl L2/3 to the DPs (originating from Cux2 positive neurons), and from ENTl L5/6 to the DPd. Refer to Extended Data Fig. 14a,b for the validation of inputs to the DP from other structures using anterograde tract tracing method. **c,** Schematic summary of neuronal inputs to the DPs and DPd from various cortical, amygdalar, and hippocampal structures. **d,** Quantitative comparison of outputs from and input to the DPs from other MPF areas like the ILA, PL, ACAv, ACAd, as well as from other cortical areas such as ORBm, MOp, and MOs (upper panel). The middle panel shows representative images of PHAL labeled axons originating from the DPs in select cortical areas shown in the bar graph (refer to Extended Data Fig. 6a,b for MPF injection sites). These axons follow two parallel projection pathways traveling through cortical layer 1 and deep layer 5, terminating densely in the ILA, PL, ACAv, ACAd, MOp, and MOs, as well as PTLp, RSPd, and visual cortices (see Extended Data Fig. 14e). The lower panel highlights the sparse distribution of retrogradely CTB labeled neurons in corresponding cortical areas from the same injection sites (see Extended Data Fig. 12a for CTB injection site in DPs). Together, this data show that DPs sends dense projections to, but receives much less inputs from, MPF and motor cortices. **e,** The schematic illustrates that the DP serves as a network junction, facilitating the transmission of neural inputs from cortical areas within the *lateral cortical network* ^38^, olfactory areas (OLF), amygdala (AMY), and hippocampus (HPF) to other MPF areas, to cortical areas within the *medial cortical network* ^38^, and to cortical motor areas. **f.** A 2D hierarchical clustering of the MPF-cortex projection fraction matrix reveals several clusters of cortical projection targets of the DPs, DPd, and other MPF areas. Prefrontal (PFC) areas are in the columns to the left of the gray line. Additional clusters are highlighted with colored boxes (Refer to Source Data 1 for Source data).

Retrograde tracer injections made into either the DPs or DPd revealed their distinct cortical inputs (Fig. 2; 3a–c; **Extended Data Figs. 12–13**). Anterograde tracer injections into upstream source structures confirmed their axonal projections to the DP (Fig. 3b; **Extended Data Fig. 14a**). Notably, cortical inputs to the DPs and DPd can be categorized as follows: (1) Both DPs and DPd receive dense inputs from the olfactory cortical areas and cortical amygdalar areas. DPs receives much denser inputs from the PIR and EPd, which processes main olfactory information, while the DPd, but not DPs, receives substantial input from the COApm and moderate input from the posterior amygdalar nucleus (PA) (Figs. 2a–f, 3a; **Extended Data Figs. 12–13, 14a**). The COApm and PA receive pheromonal information directly from the accessory olfactory bulb (AOB) and are involved in sexual and social behaviors ^47,48^. (2) Inputs from the ENTl: Notably, DPs receives inputs primarily from ENTl L2/3, while ENTl L5 neurons (not L2/3) robustly project to DPd, as well as to ILA ^38^ (Fig. 3a–c; **Extended Data Figs. 12–13**). (3) Inputs from three areas near the rhinal fissure including the temporal association (TEa), ectorhinal (ECT), and perirhinal (PERI), which together are termed as the rhinal cortex ^49^. These receive multimodal inputs from somatosensory and motor cortical areas ^38^ and generate direct projections to both DPs and DPd (Fig. 3a,c; **Extended Data Figs. 12–13, 14a**). (4) Inputs from the AUDv (Figs. 1b, 3a; **Extended Data Figs. 2a, 12–13, 14a**), responsible for processing auditory information associated with fear conditioning ^50^ are primarily to DPs. (5) The claustrum (CLA), which projects to the DPd, but not DPs, shares massive bidirectional connections with the ILA and PL ^38^ (Fig. 2g–h; **Extended Data Figs. 11g-h, 12–13**). (6) Inputs from the subiculum and CA1. Consistent with our previous work ^43^, anterograde tracer injections into the prosubiculum (ProSUB) resulted in dense axonal terminals in the DPs (Fig. 1c), while anterograde tracer injections into the ventral subiculum (SUBv) and CA1 resulted in axonal terminals specifically in DPd and ILA ^43^ (Fig. 3a; **Extended Data Figs. 12, 14a**). (7) Fear conditioning inputs: The BLAa plays a critical role in fear conditioning ^13,51^. The BLAal and BLAp, which integrates olfactory and visceral information, project to the DPs, while the BLAac and BLAam projects to the DPd and the ILA ^28^ (Fig. 1a; **Extended Data Figs. 2e-f, 12, 14b**). (8) Inputs from the agranular insular areas (AId, AIv, AIp), which process gustatory and visceral sensory information ^52,53^, project to DPd, DPs, and ILA (Fig. 3a,c; **Extended Data Figs. 12–13, 14a**). Other MPF areas, namely the ILA, PL, and ACA, provide relatively lighter recurrent projections to the DP (see below). (9) Unlike the DPs, DPd also receives inputs from several subcortical structures including, but not limited to, the lateral septum (LS) (**Extended Data Fig. 12c**).

#### DP projections to other MPF and cortical areas.

The DPs and DPd generate only minor projections back to regions that heavily innervate the DP. Instead, the DPs projects densely to other MPF areas, including the ILA, PL, ACAv, and ACAd, which send only light projections back to the DP, with the exception of the ILA (Fig. 3d–f; **Extended Data Fig. 12**). This is atypical of MPF regions, which are mostly reciprocally connected (**Extended Data Fig. 14c-d**). Additionally, the DPs project significantly to domains in the MOs and MOp that control movements of the whiskers, upper limbs, and body trunk, as well as to the dorsal retrosplenial (RSPd), posterior parietal (PTLp), and anteromedial visual (VISam) areas (Fig. 3d–f; **Extended Data Fig. 14e**). These cortical structures share reciprocal connections with the ACAd, ACAv, PL, and ILA ^38^, but send almost no projections to the DP (**Extended Data Fig. 15a-d**). See **Supplementary Results** for more details regarding quantitative comparison of MPF connectivity with other cortical areas.

Taken together, the DP mediates a predominantly unidirectional cortical information flow: it receives extensive convergent inputs from olfactory areas, amygdala, hippocampus, ENTl, and other areas along the lateral cortical mantle such as AUDv and AIp, and in turn, projects densely to other MPF and cortices along the medial edge of the neocortex (Fig. 3e). This is a distinct DP feature unique from other MPF components (**Extended Data Figs. 14c-d, 15a-d;** See additional details in **Supplementary Results**, *DP as a unique network junctional node that bridges a unidirectionally predominant cortico-cortical information flow*).

Supporting this notion, TVA receptor-mediated cell type-specific rabies viral tracing demonstrated that DPs→ACA projecting neurons receive monosynaptic inputs from the AON, PIR, SUB, CA1, and ENTl (**Extended Data Fig. 14f**). These findings further highlight that DPs and DPd exhibit distinct cortical connectivity features compared to other MPF components.

### DP receives autonomic, social behavior, spatial navigation, and conditioned memory information through its connections with midline thalamic nuclei

The MPF is known for its reciprocal connections with the mediodorsal thalamic nucleus (MD) as well as other medial and midline thalamic nuclei ^4,54,55^, although the thalamic connectivity of the DP has not been investigated. Comparative distributions of thalamo-cortical projection neurons and cortico-thalamic projection terminals for MPF regions are displayed in connectivity maps (**Extended Data Fig. 16a**; also see online map) and quantitative analyses of all thalamic connectivity are visualized in matrices (**Extended Data Figs. 16b, 17;** see **Supplementary Results:** *MPF connections with thalamus*).

The DPs receives extensive input from the RE (**Extended Data Fig. 16b**), which accounts for 76.79% of its total thalamic inputs. RE→DP projections were validated through RE anterograde tracer injections, which labeled dense axonal terminals in the DP layer 1 (Fig. 1b; **Extended Data Fig. 2c**). The DPs does not provide significant thalamic projections given its lack of cortico-thalamic (CT) neurons (**Extended Data Fig. 16a,b**). In contrast, the DPd bidirectionally connects with several midline and medial thalamic nuclei, including the RE, paraventricular (PVT), parataenial (PT), medial part of the mediodorsal (MDm), rhomboid (RH), and intermediodorsal (IMD) thalamic nuclei (**Extended Data Figs. 16c, 17a-b**). Our hierarchical clustering-based quantitative analysis revealed that DPd and ILA share similar thalamic connections specifically with the RE, PT, PVT, and MDm, while the PL, ACAd, and ACAv demonstrate distinct connections with different clusters of thalamic nuclei (**Extended Data Figs. 16e, 17a-b**). Refer to **Supplementary Results,** *MPF connections with thalamus*, for additional details regarding quantitative analysis.

Next, anterograde/retrograde tracer co-injections were made into the PVT, PT, or RE (**Extended Data Figs. 16f, 18a-b**), which validated thalamo-cortical and cortico-thalamic connections, but also identified information relayed to the DP and ILA through these thalamic nuclei. The PVT is bidirectionally connected with structures that form a network that regulates visceral sensorimotor activities (**Extended Data Figs. 16f,g, 18b**). These include (1) the parabrachial nucleus (PB) and nucleus of the solitary tract (NTS), the primary structures receiving and processing visceral sensory inputs. Notably, the PB also generates direct projections to ILA (**Extended Data Fig. 19**) ^56^. (2) the MEPO, which processes information related to fluid balance, thirst, and cardiovascular function ^57,58^. (3) The agranular insular areas (AId, AIv, AIp), central nucleus of the amygdala (CEA), bed nuclei of the stria terminalis (BST), and periaqueductal gray (PAG), all of which are involved in autonomic function control ^59,60^.

In contrast, the RE and PT bridge the DP and ILA with the hypothalamic networks that govern two fundamental types of social behavior (namely reproductive and defensive) as well as with structures implicated in spatial navigation and conditioned memory (**Extended Data Figs. 16f,g, 18a,b**). Given that the RE is a primary source of thalamic input to DPs and DPd, we applied a TVA receptor-mediated rabies tracing technique to further disclose which structures provide monosynaptic inputs to the RE→DP neurons (**Extended Data Fig. 18c-d**). Importantly, prefrontal-RE pathways have been implicated in the regulation of emotions, including contextual fear ^61^ and emotional memory related to predator exposure ^62^.

### DP projects to structures that regulate autonomic, neuroendocrine, and behavioral activities important for maintaining homeostasis and regulating social behavior

#### DP projections to cerebral nuclei that control visceral motor activities.

All neocortical areas, including MPF areas, except for the DP, project heavily to the caudoputamen (CP, dorsal striatum in rodents) ^42^. Instead, the DPs exhibits remarkably dense projections to the central amygdalar nucleus (CEA) and substantia innominata (SI), and relatively light projections to the anterolateral division of the BST (BSTal) (**Extended Data Fig. 20a-e**), all of which are extensively interconnected as a core network that regulates autonomic function ^60,63^. In contrast, the DPd densely projects to anteromedial BST (BSTam) (**Extended Data Fig. 20a, d-e**), which regulates neuroendocrine activities of the hypothalamic-pituitary-adrenal axis (HPA) in response to stress ^1,64–66^. Notably, the DPd and ILA provide significantly weaker projections to the CEA and instead, topographically project to the MEA, posterior division of the BST (BSTp), and hypothalamic medial nuclei (**Extended Data Fig. 20a,c,e**), which form two parallel subnetworks that govern reproductive and defensive behaviors ^45,46,48,59,67,68,69^. Like the ILA, the DPs and DPd also topographically project to the olfactory tubercle (OT) and nucleus accumbens (ACB) (**Extended Data Fig. 20b-d**) well-known for their roles in reward and social interactions ^70,71^.

#### DP projections to preautonomic structures in the hypothalamus and brainstem.

The MPF coordinates autonomic, neuroendocrine, and behavioral responses crucial for maintaining emotional equilibrium ^1–3^. Despite a partial understanding of the underlying neural circuits, our systematic and comprehensive mapping reveals that different MPF areas generate topographic projections to the hypothalamus (**Extended Data Fig. 21a**) and brainstem structures. For additional details regarding *topographic projections from MPF to LHA*, Refer to **Supplementary Results.**

##### Hypothalamic preautonomic structures.

**Figure 4:**
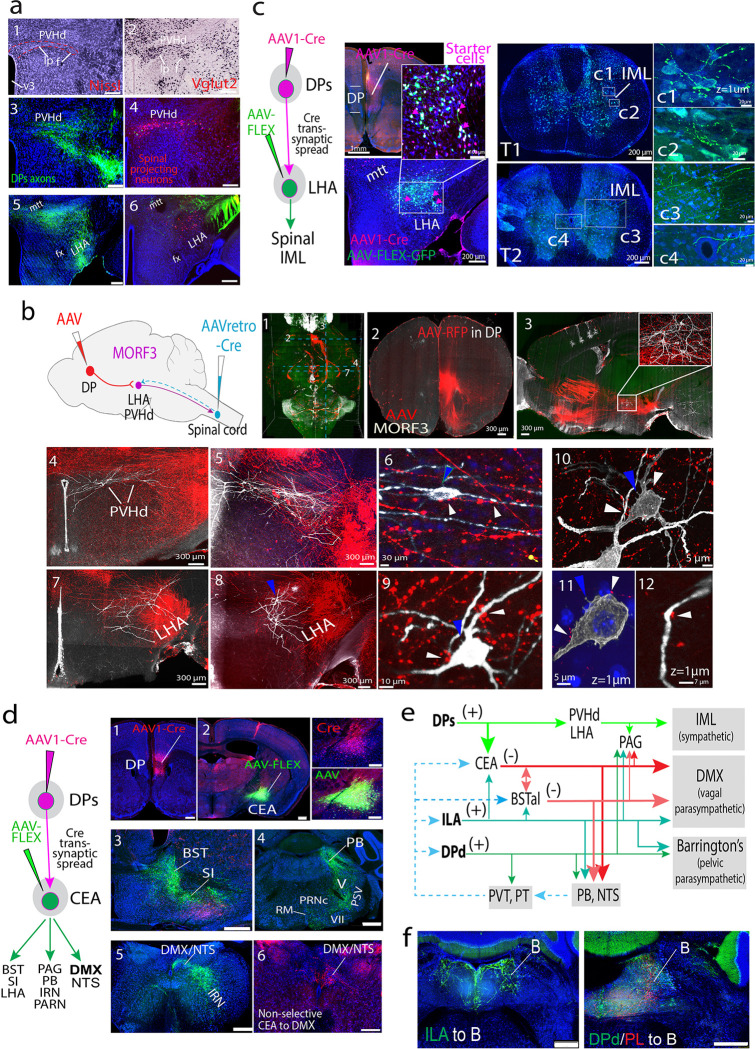
DP projections to brain structures in controlling autonomic outputs **a,**PHAL labeled axons originating from the DPs innervate two descending parts of the PVH (PVHd), namely the lateral parvicellular and forniceal parts (PVHlp and PVHf) (**a1-a3**), which contain spinal projecting neurons revealed by a CTB injection into the spinal cord (**a4**). Corresponding Nissl stained image (**a1**) validates cytoarchitecture of the PVHlp and PVHf, which contain VGLUT2 positive neurons (**a2**, image from Brain-map.org). Refer to **Extended Data Fig. 21a**for PHAL injection site in DPs. DPs axons densely distribute in the LHA (**a5**), which also contains CTB labeled spinal projecting neurons (**a6**). All scale bars = 200 μm. **b,**Our strategy of uncovering potential synaptic connections between DP axons and hypothalamic spinal projecting neurons (upper left panel). In MORF3 mice, we injected AAV-RFP into the DP (see **b1, b2** for injection site) to label its axons, and AAVretro-Cre into the spinal cord. Cre was retrogradely transported to the hypothalamus, triggering the expression of MORF3 and allowing spinal projecting neurons to display finely detailed dendritic morphology (**b3**). High-resolution 3D lightsheet (**b1–4, b7**) and confocal (**b5–6, b8–12**) microscopy images reveal numerous close appositions between DP axonal terminals and the somas and dendrites of MORF3 labeled neurons in the PVHd (**b6**) and LHA (**b7–12**) at higher magnifications. Blue arrowheads point to corresponding neurons at different magnifications. White arrowheads highlight close appositions between DP axonal terminals and spinal projecting neurons (see **Supplementary Video 1** for multiple putative synaptic connections between the same DP axon and a MORF3 labeled neuron). **c,**Using a combined AAV1-Cre transsynaptic tagging and Cre-dependent anterograde tracing method, we confirmed that LHA neurons receiving direct DP inputs generate direct projections to the intermediolateral column (IML) of the spinal cord, which houses preganglionic neurons that control sympathetic output. AAV1-Cre was injected into the DP (upper left panel) to anterogradely transport and trans-synaptically spread into postsynaptic LHA neurons, which received an injection of Cre-dependent AAV-FLEX-GFP (lower left panel). This led to the labeling of starter cells generating axonal projections that innervate preganglionic neurons in the IML, as depicted in the microscopic images at thoracic T1 and T2 levels of the spinal cord. The close-up views in the right panels provide a detailed observation of AAV labeled axons, displaying terminal boutons forming close appositions onto IML neurons (**c1, c2, c3**) and around the center canal (**c4**). **d,** Our strategy of uncovering a bi-synaptic pathway through which the DPs regulates vagal parasympathetic output via its projections to the CEA. AAV1-Cre was injected into the DPs (**d1**). Subsequently, Cre was anterogradely transported to the CEA, which received an injection of Cre-dependent AAV-FLEX-GFP (**d2**) to visualize axonal projections originating from Cre labeled postsynaptic neurons (**d3–5**). Close up images illustrate starter cells in the CEA, colabeled with Cre and AAV-GFP (**d2**). Dense axonal terminals labeled with AAV-GFP were observed in the DMX and NTS (**d5**), confirming the bi-synaptic pathway DP→CEA→DMX. Additionally, dense axonal labeling was evident in other brain structures involved in autonomic function control, including the bed nucleus of the stria terminalis (BST, **d3**), substantia innominata (SI, **d3**), parabrachial nucleus (PB, **d4**), parvicellular reticular nucleus (PARN), and intermediate reticular nucleus (IRN, **d5**). The lower right image shows axonal terminals resulting from non-selective CEA neurons in the DMX/NTS as a comparison (d6). All scale bars = 500 μm. **e**, Schematic illustrating that the DPs, DPd, and ILA form a core cerebral neural network in controlling autonomic outputs (refer to Extended Data Figs. 16–21, 23–24 for additional data). **f,** Direct projections to Barrington’s nucleus (B) from the ILA (left) and DPd (green) (right panel), indicating bi-synaptic connections of the MPF in controlling lumbosacral parasympathetic outputs (DPd/ILA→Barrington’s nucleus→spinal cord). Scale bars = 500 μm.

The DPs exhibits extensive projections that directly innervate the descending parts of the hypothalamic paraventricular nucleus (PVHd), namely the lateral parvicellular (PVHlp) and forniceal parts (PVHf) (Fig. 4a1,a3, **Extended Data Fig. 21a,b**). These regions contain VGLUT2 positive preautonomic neurons that send descending projections to the spinal cord's intermediolateral (IML) column (Fig. 4a2, a4), which controls sympathetic outputs ^59,72,73^. In addition, the DPs generates dense projections to the LHA (**Extended Data Figs. 21a,c, 22a, 23a**), which also contains preautonomic neurons that project to the spinal cord (Fig. 4a5–a6), suggesting that DPs neurons likely innervate spinal cord projecting LHA neurons.

To test this, in MORF3 mice ^34^, AAV-RFP was injected into the DP to label its axonal projections, while AAVretro-Cre was injected into the spinal cord (Fig. 4b1–b2). Cre was retrogradely transported to spinal projecting neurons and stochastically unlocked MORF3 expression (Methods). As expected, this revealed the dendritic morphology of MORF3 labeled PVHd and LHA neurons, which were heavily intermingled with axonal terminals arising from the DP (Fig. 4b3–b5, b7–b8). Despite MORF3 labeled neurons accounting for only a small fraction (3–5%) of total spinal projecting neurons due to the sparse labeling properties of MORF3 technology ^34^, significant numbers of close appositions were observed between DP axonal terminals and MORF3 labeled somas and dendrites (Fig. 4b6, b9–b12; **Supplementary Video 1**), indicating putative synaptic connectivity through which DP innervates LHA-spinal projecting neurons.

Next, by employing AAV1-Cre anterograde transsynaptic tagging alongside Cre-dependent AAV anterograde tract tracing ^74^, we confirmed that the postsynaptic DP-input-recipient neurons in the LHA project directly to IML preautonomic neurons in the spinal cord (Fig. 4c; also see **Extended Data Fig. 23b1–3** for additional injection information). Supporting this finding, our data showed that those DP-input-recipient neurons in this LHA region generate very sparse projections to the DP, thus, confirming DP→LHA connections are primarily unidirectional (**Extended Data Figs. 22a, 23b4**). Meanwhile, those DP-input-recipient LHA neurons also generate dense projections to the SI, lateral habenular nucleus, and brainstem structures such as PAG, PB, and nucleus raphe magnus (RM) (**Extended Data Fig. 23b5–19**), all of which are directly involved in autonomic function. See **Supplementary Results** for additional details on how *DP projects to hypothalamic preautonomic structures*.

##### Brainstem autonomic connections.

In rats ^35,36^, ILA sends direct excitatory projections to the dorsal motor nucleus of the vagus nerve (DMX), which controls vagal parasympathetic output. Our findings in mice confirmed that ILA, but not DP, projects directly to DMX (**Extended Data Fig. 23c**). Instead, the DPs projects to CEA (**Extended Data Fig. 20a,c,d**), which in turn projects to DMX. Using AAV1-Cre transsynaptic tagging combined with Cre-dependent AAV-GFP anterograde tracing, we verified that CEA neurons receiving direct inputs from DP display dense axonal terminals in the DMX (Fig. 4d; DP→CEA projections are unidirectional, see **Extended Data Fig. 22b**). Considering the CEA's GABAergic nature, these data establish a bi-synaptic pathway, DPs→CEA→DMX, through which the DP can inhibit vagal parasympathetic outputs. Notably, DP-input-recipient CEA neurons generate extensive projections to the BSTal, PB, and NTS (Fig. 4d), all of which also project to the DMX ^63^. The PB and NTS are primary brainstem structures that process visceral sensory information. While the ILA generates dense projections to both structures, the DPd also project to the PB (**Extended Data Fig. 19**) ^56^. Together, the ILA, DPs, CEA, BSTal, PB, and NTS form a core network that regulates vagal parasympathetic output (Fig. 4e; **Extended Data Fig. 23d**).

Further, the ILA and DPd project to Barrington's nucleus (Fig. 4f), which directly innervates parasympathetic motor neurons in the lumbosacral regions of the spinal cord that generate the pelvic nerves to influence micturition, defecation, and penile erection ^75,76^. Finally, it is important to mention that, like other MPF components ^4^, the ILA, DPs, DPd, and other MPF areas generate topographic projections to the PAG (**Extended Data Fig. 24a-c**), through which they can regulate autonomic and behavioral outputs. See **Supplementary Results** for additional details on MPF connections with PAG.

#### Direct projections of the DPd to the hypothalamic neuroendocrine zone.

**Figure 5:**
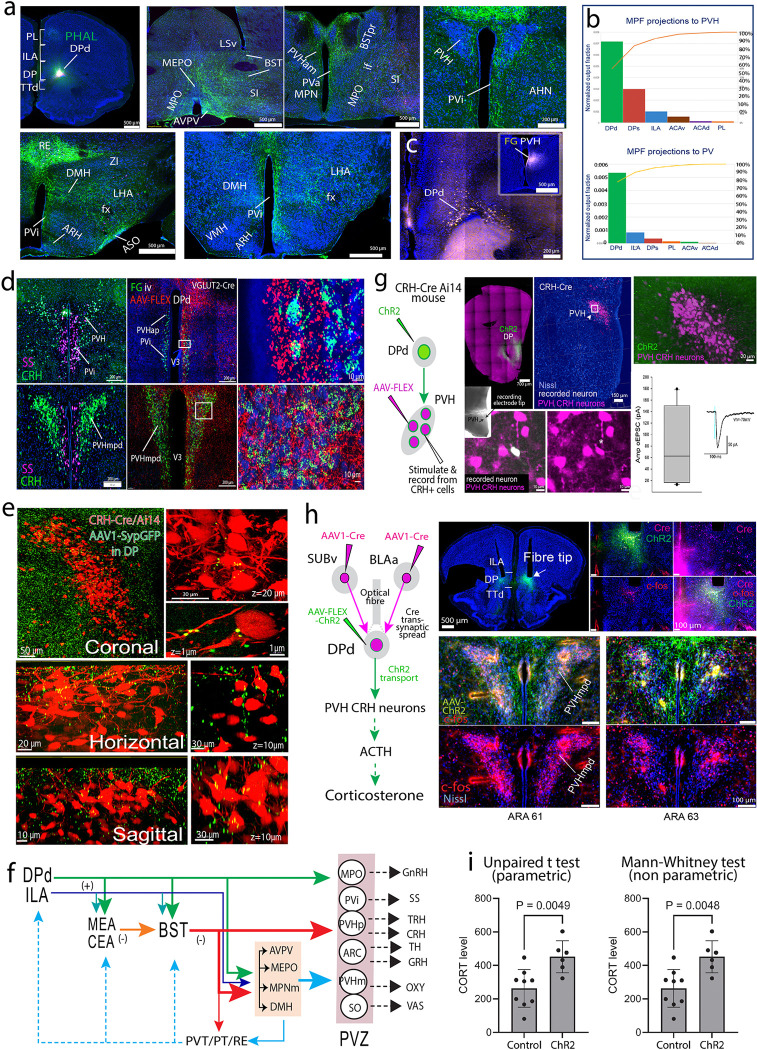
DPd projections to the hypothalamic neuroendocrine zone. **a,** Images show axonal projections originating from the DPd generating dense axonal terminals along the entire hypothalamic periventricular zone, which houses the vast majority of neuroendocrine cells (refer to Extended Data Fig. 25 for additional data). See Table 1 for additional abbreviations of brain structures. All scale bars = 500 μm. **b,** Quantitative comparison of projections from different MPF areas to the paraventricular hypothalamic nucleus (PVH), which contains both neuroendocrine and pre-autonomic cells, and the periventricular nucleus (PV), primarily composed of neuroendocrine cells. The pareto chart, which orders the number of projections in descending order showed that both the PVH and PV receive the densest input from the DPd (also see Extended Data Fig. 25b-c). The cumulative total of projections is represented by the curved line. Projections from the DPs to the PVH likely target the PVH descending part, which contains preautonomic neurons as shown in Fig. 4a. **c,** Shows retrogradely labeled neurons in the DPd after FG was injected into the PVH, validating the direct DPd→PVH projections. Refer to Extended Data Fig. 26a for further validation of monosynaptic DPd inputs to PVH CRH neurons using TVA-receptor mediated cell type-specific rabies viral tracing method. **d,** To further validate specificities of DPd projections to the hypothalamic neuroendocrine zone, in VGLUT2-Cre mice, AAV-FLEX-RFP was injected into the DPd (see Extended Data Fig. 26a for injection site; see Fig. 1d for Vglu2 expression specificities in the DPd). VGLUT2 positive neurons in the DPd produced dense projections that directly innervated hypothalamic neuroendocrine neurons, which are labeled with retrograde tracer fluorogold (FG, through a tail vein injection, middle and right panels). Using RNAscope (left panel), we confirmed the distribution of somatostatin (SS) neuroendocrine neurons in the periventricular nucleus (PVi) and periventricular part of the PVH (PVHpv), as well as corticotropin-releasing hormone (CRH) neurons in the PVHmpd. DP axonal terminals are densely intermingled with those neuroendocrine cells and form close appositions, as depicted in the close up images (right panel) (see Extended Data Fig. 25d for location of different neuroendocrine cells). **e,** Shows putative DP connections onto PVH CRH neurons. An AAV synaptophysin tagged GFP anterograde tracer was injected into the DP of CRH-Cre/Ai14 mice. High resolution 60x images show terminal from the DP (green) contacting CRH neurons in the PVH (red). These putative synaptic contacts can be clearly seen in the coronal, horizontal, and sagittal views. **f,** Schematic network model illustrating how the DP, along with the MEA, CEA, and BST, regulates hypothalamic neuroendocrine outputs. They achieve this through direct projections to the hypothalamic periventricular zone or indirectly via projections to various hypothalamic nuclei, such as AVPV, MEPO, MPNm, and DMH (see Extended Data Figs. 25a, 26f, 27a-b). **g,** Functional validation of monosynaptic innervation of DPd neurons onto CRH PVH neurons. The left panel illustrates the experimental strategy. In CRH-Cre mice, ChR2 was injected into the DP, while Cre-dependent AAV-RFP was injected into the PVH to label CRH positive neuroendocrine cells. This allowed examination of CRH neuron responses to optogenetic stimulation of ChR2 positive axons originating from the DP using slice patch-clamp recordings. The middle panels display ChR2 injection in the DP and CRH positive neurons in the PVH, intermingled with ChR2 positive axons. The boxed region highlights representative biocytinlabeled CRH neurons from the patch-clamp recording experiment. In the lower panels, close up images show co-localization of biocytin (left) and CRH (right) in the same neuron. The box-and-whisker plot on the right depicts the distribution of peak amplitude responses evoked by optogenetic stimulation of DP inputs (12/14 neurons responded), indicating the median, highest, and lowest values. The trace on the right is a representative example of a response from a CRH neuron. Median peak amplitudes of currents evoked by optogenetic stimulation were not significantly different between groups (83±21 pA, n=14). **h-i,** Experimental design to demonstrate the functional regulation of the HPA axis by DPd in response to inputs from the ventral subiculum (SUBv) and BLAa. **h,** Following the injection of AAV1-Cre into the SUBv and BLAa, Cre is anterogradely transported and transsynaptically spreads to the postsynaptic DPd neurons, which received an injection of Cre-dependent AAV-FLEX-ChR2. Consequently, only those neurons that receive direct inputs from the SUBv and/or BLAa express ChR2. This ChR2 is then anterogradely transported to the PVH and innervates CRH neurons, which control the HPA axis. Therefore, optogenetic stimulation of DPd neurons should result in elevated plasma corticosterone (CORT) levels. The right panel presents microphotographs showing the fiber tip and the corresponding expression of Cre, ChR2, and c-fos. The lower panel illustrates the PVHmpd containing ChR2 axons originating from the DPd and the increased expression of c-fos in response to optogenetic stimulation. **i**, Plasma CORT levels are significantly higher in the ChR2 group compared to the control group, which received DP injections of AAV-mCherry. The difference is statistically significant, with p=0.0049 in the unpaired t-test (parametric) and p=0.0048 in the Mann-Whitney test (nonparametric).

The prevailing understanding is that the MPF (ILA, PL) does not directly innervate hypothalamic neuroendocrine cells, but instead regulates neuroendocrine activities indirectly through the BST and other intermediary structures ^66,77^. Our data support this by confirming that ILA and PL do not directly project to the hypothalamic neuroendocrine zone. However, we reveal the novel finding that DPd sends direct and extensive projections to the entire hypothalamic neuroendocrine zone along the third ventricle (Fig. 5a–b; **Extended Data Fig. 25a-d**). This zone encompasses motor neuron pools consisting of various parvo-and magnocellular neurosecretory neurons ^59,68,72^, including (1) the septal and rostral ends of the medial preoptic region (MPO) contain gonadotropin-releasing hormone (GnRH) motor neurons; (2) the anterior periventricular nucleus (PVa), parvicellular neuroendocrine part of the paraventricular nucleus (PVHp), and arcuate nuclei (ARH) house parvicellular neuroendocrine cells including somatostatin (SS), corticotropin-releasing hormone (CRH), thyrotropin-releasing hormone (TRH), growth hormone-releasing hormone (GRH), and dopamine (DA, which inhibits prolactin release); (3) the magnocellular neuroendocrine part of the PVH (PVHm), supraoptic (SO), and accessory supraoptic (ASO) nuclei contain magnocellular oxytocin and vasopressin neuroendocrine cells (**Extended Data Fig. 25d** shows the distribution of these neuroendocrine neurons depicted on a mouse hypothalamic flatmap).

Next, retrograde FG and CTB tracer injections in the PVH (Fig. 5c) and ARH (**Extended Data Fig. 25e**), confirmed these connections and showed retrogradely labeled neurons only in DPd and not in ILA or other MPF areas. Next, using VGLUT2-Cre mice, we showed that DPd VGLUT2 positive neurons directly innervate hypothalamic parvocellular and magnocellular neuroendocrine cells, which we labeled through the intravenous injection of Fluorogold (FG) ^72^. Axon terminals originating from VGLUT2 DPd neurons intermingled with FG labeled cells in the PVH, SO, and periventricular nucleus (PV) (Fig. 5d; **Extended Data Fig. 26a-c**), suggesting putative synaptic connections between the DPd axonal terminals and these neuroendocrine cells. Employing RNAscope in the same animals, we confirmed that the preoptic (PVpo) and anterior periventricular (PVa) nucleus and the periventricular part of the PVH (PVHpv), whose neurons synthesize SS, receive the densest inputs from the DPd (Fig. 5d). Importantly, the dorsal medial parvicellular part of the PVH (PVHmpd), which contains the majority of CRH- and TRH-synthesizing neurons, receives substantial input from DPd VGLUT2-expressing neurons (Fig. 5d; **Extended Data Fig. 26a**). To confirm the direct innervation of DP axonal inputs onto CRH neurons, we injected an AAV1-synaptophysin-GFP anterograde tracer into the DPd, labeling its axonal terminals in the PVH of CRH-Cre/Ai14 mice, where CRH neurons are marked by tdTomato expression (Fig. 5e). High-resolution (60x) confocal imaging of the PVH revealed numerous GFP-labeled terminal boutons in close apposition to CRH neurons (Fig. 5e). Furthermore, cell-type-specific monosynaptic rabies tracing (TRIO) in CRH-Cre mice identified back-labeled neurons in the DPd, confirming direct monosynaptic input from the DPd to PVH CRH neurons (**Extended Data Fig. 26d**; see Methods).

Within the arcuate nucleus (ARH), a highly dense terminal plexus is concentrated dorsomedially (Fig. 5a; **Extended Data Fig. 26c**), where dopamine neuroendocrine motoneurons are located. Comparatively, lighter inputs from DPd were observed in the ventrolateral regions of the ARH, where neurons synthesizing growth hormone-releasing hormone (GRH) predominate. Immunostaining confirmed that DPd axons form putative synaptic connections with magnocellular oxytocin positive neurons in the PVH and SO (**Extended Data Fig. 26e**).

As aforementioned, the DPd densely innervates the BSTam (Fig. 5a; **Extended Data Figs. 20a, e, 25a, 26f**). In turn, the BSTam provides extensive projections to the entire hypothalamic neuroendocrine zone (**Extended Data Fig. 26f**) ^64^. Both the DPd and BSTam also send dense projections to several other hypothalamic structures, including the anteroventral periventricular (AVPV), median preoptic (MEPO), medial preoptic (MPN), and dorsomedial hypothalamic nuclei (DMH), as well as the medial preoptic area (MPO) (Fig. 5a; **Extended Data Fig. 25a, f-g**). These structures, which are components of the hypothalamic visceral motor pattern generator ^59^, densely project to the hypothalamic neuroendocrine zone (**Extended Data Fig. 27a**) and other structures involved in the regulation of autonomic function (e.g., PVHlp, LHA, PAG, PB, and Barrington’s nucleus) and social behavioral activities (e.g., VMH, PMv, and PMd) ^59^. Consequently, the DPd, BSTam, and these hypothalamic structures organize into a core network that regulates neuroendocrine motor function (Fig. 5e; **Extended Data Fig. 27b**). Refer to the Supplementary Results for additional details about *DPd connections with neuroendocrine structures.*

### Functional demonstration of DP neurons directly regulating neuroendocrine responses

We validated functional monosynaptic connections from the DPd onto CRH neuroendocrine cells by performing single-cell electrophysiological recordings using channelrhodopsin-assisted circuit mapping (CRACM) combined with anterograde transsynaptic tracing (Fig. 5g). Cre-dependent AAV1 expressing tdTomato was injected into CRH-Cre mice to label CRH neurons in the PVH, while AAV-ChR2-YFP was injected into the DPd to label its axonal projections. After 4 weeks, 350 μm brain slices containing the PVH were prepared to patch and record from tdTomato labeled CRH neurons in the PVH, with optical stimulation of ChR2 labeled axonal terminals originating from the DP. Following recording, labeled neurons were infused with biocytin, and the brain slices were PFA-fixed and immunostained to reveal biocytin labeled recorded neurons (see Methods). Low-magnification images of the slices captured the distribution of tdTomato positive CRH neurons and GFP (channelrhodopsin)-expressing axons from the DP, while high-magnification images detailed the anatomical locations and morphology of recorded neurons. The data showed that 12 out of 14 tdTomato labeled cells in the PVH responded to channelrhodopsin stimulation with blue light, with an average response amplitude of 83±21 pA (Fig. 5g), confirming monosynaptic inputs from the DP onto CRH neurons in the PVH.

Next, we explored which neural inputs might drive the DPd's outputs to regulate motor outputs of CRH neuroendocrine cells as indicated by changes in corticosterone (CORT) levels. Specifically, we focused on two upstream structures: the ventral subiculum (SUBv), which is associated with psychological stress (e.g., restraint stress) and emotional regulation ^1,78^, and the BLAa implicated in fear conditioning ^79^. Utilizing a combination of AAV1-Cre anterograde transsynaptic tagging and multi-fluorescent anterograde tract tracing techniques in MORF3 mice, we revealed that postsynaptic DP neurons that receive inputs from SUBv and BLAa, project extensively to the PVH (**Extended Data Fig. 28a-h**). Note the unidirectional BLA/SUBv→DP connection (**Extended Data Fig. 22d-f**). Our findings also suggest that inputs from SUBv and BLAa potentially converge onto the same DPd projection neurons (**Extended Data Fig. 28i-n**). Altogether, these data validate a bi-synaptic SUBv/BLAa→DPd→PVH circuit pathway.

To functionally confirm this hypothesis, we administered AAV1-Cre into the SUBv and the BLAa, and Cre-dependent AAV-ChR2 into the DP. As a result, only postsynaptic DP neurons that receive inputs from SUBv and/or BLAa would express ChR2 and send downstream projections to the PVH (Fig. 5h). Optogenetic activation of these specific DP postsynaptic neurons led to a significant increase in plasma CORT levels compared to the control group (p=0.0049 unpaired t test; p=0.0048, Mann-Whitney test) (Fig. 5i). This evidence of a functional circuit suggests that DP neurons can directly modulate plasma CORT levels (and potentially other neuroendocrine activities) in response to signals from the SUBv and BLAa. Consistent with our results, a recent study also showed that chemogenetic stimulation of DP/TTd resulted in increased plasma CORT levels ^21^.

### Defining the functional relevance of DP neurons in integrating information from external and internal environments for social and non-social responses

Our connectome data reveal that the DP receives a diverse array of cortical and thalamic inputs and, in turn, regulates neuroendocrine, autonomic, and behavioral responses to environmental stimuli. To further validate this hypothesis and explore its functional relevance, we conducted the following studies.

#### DP neurons receive convergent sensory and contextual memory synaptic inputs.

To our knowledge, the DP is the only prefrontal area to receive converging inputs from olfactory (PIR, EPd), auditory (AUDv), and entorhinal (ENTl) cortices (Fig. 1b, **Extended Data Fig. 2a-b, d**). The integration of inputs from these three cortical areas is significant because olfactory and auditory signals are important social cues and for responding to predators, while the ENT provides spatial and contextual memory information ^48,50,80,81^.

**Figure 6:**
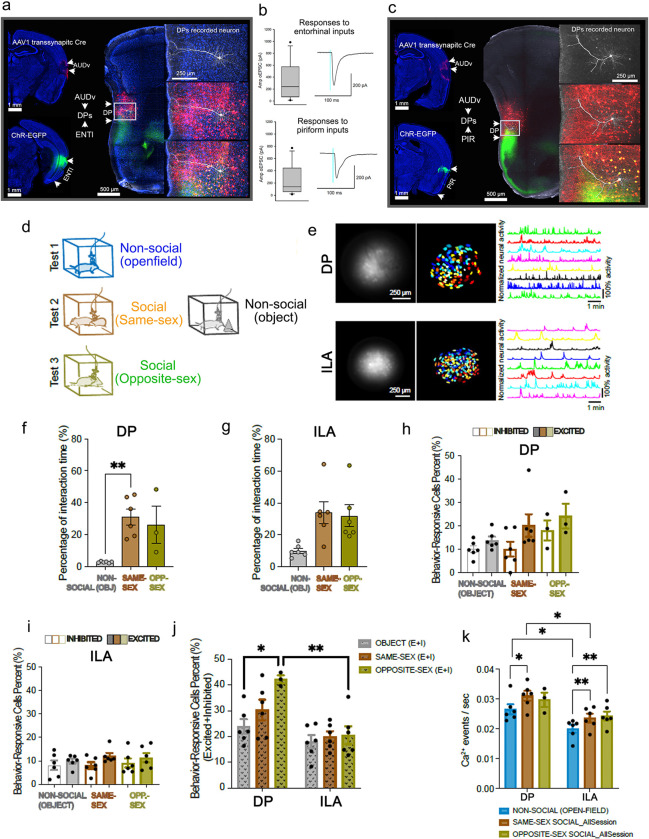
Functional relevance of DP neurons in integrating different sensory modalities and regulating social behavior **a-c,** Convergent inputs from the AUDv and ENTl or AUDv and PIR onto DP neurons. **a, c,** Left panels showing images of AAV1-Cre injections into the AUDv and AAV-ChR2 into either ENTl (**a**) or PIR (**c**). In both cases, Cre-dependent AAV-RFP was injected into the DP to reveal postsynaptic neurons transported from the AUDv, which are intermingled with ChR2 positive axons arising from the ENTl (**a**) or PIR (**c**). The close up images show representative biocytin labeled DP neurons from patch-clamp recording experiments. **b,** Shows patch clamp recording results. The box and whisker plot on top shows the distribution of peak amplitude responses evoked by optogenetic stimulation of ENTl inputs (12/17 neurons responded). The median, highest, and lowest values are indicated. Trace on the right is a representative example of a response from an RFP-tagged neuron in DP. The plot on the bottom shows distribution of peak amplitude responses evoked by optogenetic stimulation of piriform cortex inputs (10/11 neurons responded) and a representative trace example. Median peak amplitudes of currents evoked by optogenetic stimulation were not significantly different between groups (137.4 pA, n=11 from piriform cortex and 218.3 pA, n=12 from entorhinal cortex, p=0.782, Mann-Whitney Rank Sum Test) (see Extended Data Fig. 29a for procedure to isolate monosynaptic responses). **d-i,** DP and ILA imaging during social behavior. **d,** Behavioral schematic: miniscope calcium imaging during non-social interactions (open-field and object exploration) and social interactions (same-sex partner and opposite-sex partner) synchronized with behavior camera recording. **e,** Calcium imaging field of view and example extracted traces (top: DP, bottom: ILA). **f-g,**Percentage of time miniscoped-animals spent interacting with non-social (object) versus social (same-sex, opposite-sex) targets during the behavioral trials. Interaction time was significantly different across different type of interactions (Left, DP; Right, ILA). One-way ANOVA: DP, F(1.473, 5.154)=13.47, *P=0.0108; ILA, F(1.646, 8.230)=6.883, P=0.0205. Tukey's multiple comparisons test: **P=0.0051. Error bars represent standard errors of the mean. **h-i,**Percentage of behaviorally responsive cells during interactions with non-social stimuli (object) or social target (same-sex or opposite-sex). **j,**Percentage of all behaviorally responsive cells (Inhibited+Excited). DP contains significantly more opposite-sex interaction responsive cells than object exploration responsive cells. Tukey's multiple comparisons test: *P=0.0149. Significantly more opposite-sex interaction responsive cells are identified in DP than in ILA. Sidak's multiple comparisons test: **P=0.0027. See Extended Data Fig. 29b-c for additional data. **k,** Frequency of calcium events during non-social behavior trials (open-field) and social behavior trials (same-sex and opposite-sex). In DP, frequency of calcium event is significantly higher during same-sex interaction compared to that during open-field test (Tukey's multiple comparisons test, *P=0.0303). In ILA, frequencies of calcium event during both same-sex interaction (Tukey's multiple comparisons test, **P=0.0086) and opposite-sex interaction (Tukey's multiple comparisons test, **P=0.0094) are significantly higher than that during open-field test. Between DP and ILA, frequencies of calcium event during open-field test (Sidak's multiple comparisons test: *P=0.0416) and same-sex interaction (Sidak's multiple comparisons test: *P=0.0199) are higher than that in ILA.

To functionally validate the synaptic convergence of these neural inputs onto DP neurons, we once again conducted single-cell electrophysiological recording experiments using CRACM combined with anterograde transsynaptic tracing ^44^ (Methods). We injected AAV.ChR2 into the ENTl and AAV1-Cre into AUDv (Fig. 6a). The AAV1-Cre anterogradely transported from the AUDv to DP to transsynaptically infect postsynaptic DP neurons, which expressed tdTomato. Meanwhile, ChR2 labeled axons arising from the ENTl distributed in DP layer 1. If the tdTomato labeled postsynaptic neurons also receive inputs from ENTl, we would expect them to respond to stimulation of ChR2 axons. Notably, AUDv→DP connections are unidirectional (**Extended Data Fig. 22g**). Patch clamp recordings of tdTomato labeled neurons confirmed this prediction, as nearly all DP neurons (n=10/11) responded to stimulation (Fig. 6b). This suggests that neural inputs from ENTl and AUDv converge onto the same DP neurons. Using the same strategy, we demonstrated that DP neurons (n=12/17) receive convergent auditory (from AUDv) and olfactory (from PIR) inputs (Fig. 6b–c). After recording, slices were PFA-fixed and immunostained for biocytin, which confirmed the anatomic location of the recorded cells within the DPs (Fig. 6a, c).

#### DP neurons’ role in perceiving social and environmental stimuli.

We further explored the potential functional relevance of DP neurons in social cognition and interaction – an MPF function that has been extensively studied ^5,7,12^. Social interactions necessitate the perception and integration of diverse neural inputs, including olfactory, pheromonal, auditory, visual, and somatosensory cues. These inputs undergo instantaneous modifications through dynamic, mutual feedback between participants, as well as past social experiences ^48^. The DP occupies a unique position to receive and integrate information relayed from cortical and thalamic inputs, and in turn, projects to the MEA, BST, hypothalamic medial nuclei and other structures implicated in social behavior. These connectivity data lead us to hypothesize that DP neurons play a role in perceiving and regulating social behavior.

To test this, we performed calcium imaging using miniaturized microscopy in DP during both social and non-social behavioral contexts (Fig. 6d; Methods). We expressed GCAMP6f specifically in VGLUT2 positive glutamatergic DP neurons in VGLUT2 Cre mice (Fig. 6e). In social trials, we monitored DP activity in mice engaged in interactions including chasing, grooming, or mounting, either with a same-sex or opposite-sex partner (Fig. 6f). In non-social trials, we recorded DP activity in mice within an open-field arena or during the exploration of an object (Fig. 6f). We used ROC analysis to identify DP neurons that were either significantly inhibited or excited during interactions with same-sex partners, opposite-sex partners, or objects. Among the recorded DP neurons (6 male mice, 63.3±9.9 per animal), a substantial proportion responded during either same-sex (6 male mice, 30.3±4.0%) or opposite-sex (3 male mice, 42.2±1.5%) social interactions (Fig. 6h–j). Notably, socially excited neurons outnumbered socially inhibited neurons (Fig. 6h; **Extended Data Fig. 29b**).

While we also observed that DP neurons respond to interactions with a non-social object (23.9±2.8%), the frequency of calcium events during non-social behavior trials was significantly lower compared to social behavior trials (same-sex interaction) (Fig. 6k; DP, object vs same-sex, *p=0.0303; Tukey’s multiple comparisons test).

As a control, we also examined neuronal activity in the ILA in response to the same social and non-social cues (6 male mice, 133.5+14.4 average number of ILA neurons recorded). Since the ILA lacks VGLUT2 positive neurons, we expressed GCaMP6f in VGLUT1 positive glutamatergic ILA neurons (Fig. 6e). As previously described, the ILA displays distinct input/output patterns compared to the DP, notably lacking inputs from the COApm or PA, and its projections to the hypothalamus primarily target the anterior hypothalamic nucleus (AHN), which is involved in defensive behavior ^59^. Interestingly, the ILA contains significantly less opposite-sex interaction-responsive cells (Sidak's multiple comparisons test: **P=0.0027; Fig. 6g, i,-j) and lower frequency of calcium events during non-social open-field test (Sidak's multiple comparisons test: *P=0.0416) and same-sex social interaction (Sidak's multiple comparisons test: *P=0.0199) compared to DP. (Fig. 6g, k; **Extended Data Fig. 29c**).

Consistent with these findings, using c-fos as a marker of neuronal activity, our study further suggested that DP neurons in male mice respond to the presence of both male (intruders) and female mice, as well as pups (**Extended Data Fig. 30a**). Further, our results show that DP neurons can be activated by a variety of stressors and perceived threats, including acute restraint stress, sudden looming sounds, and foot shock-based fear conditioning (**Extended Data Fig. 30b-c**). These findings, coupled with the broad range of neural inputs received by the DP, suggest its role in processing environmental cues important for social interactions and evaluation of threat. This capability is essential for assessing risks, making decisions, and preparing for appropriate behavioral responses to safeguard well-being. Consistent with this hypothesis, recent research suggests that neurons in the DP/TTd region are implicated in the psychological stress resulting from social defeat ^19,20^, top-down control of defensive behavior ^23^, in depression, anxiety, and the encoding of fear memories related to auditory cues ^21,22^, as well as opioid reward ^24^ and acquired sociopathy ^14^.

## Discussion

### Technological considerations.

This study contributes to our ongoing efforts to construct a comprehensive whole-brain mesoscale connectome of the mammalian brain. Our previous work, and those from other groups, has systematically mapped the neural networks of the cortico-basal ganglia-thalamic networks essential for motor control ^32,33,38,39,40,42,44^ and cortico-tectal systems ^45^. In parallel, we have delineated the intricate networks within the classic limbic system, including the hippocampus ^43^, amygdala ^28,82^, and hypothalamus ^72,83^, which regulate goal-directed behaviors associated with emotion. The medial prefrontal cortex (MPF) is an interface between these motor and limbic systems. The DP, ILA, and PL share connectivity with limbic regions like the hippocampus, amygdala, and hypothalamus, while the ILA, PL, and ACA also project to the basal ganglia and superior colliculus ^42,44,45^. This study’s global input/output wiring diagram of MPF represents a pivotal step toward the completion of the whole brain connectome.

MPF connectivity has been extensively studied across species using various techniques ^8^, though many studies yield fragmented views. As part of our systematic mapping of neural pathways in the mouse brain, we employed multi-fluorescent tract tracing and viral labeling, following methods in our previous work ^38,42,44^. While each tracer type has unique properties and neurotropism (for details, see corresponding tracer subsections in Methods), all injections underwent rigorous validation through qualitative assessment and cross-verification (e.g., **Extended Data Figs. 2, 10, 11, 14, 19, 26**) ^28,38,42,44^. Retrograde and anterograde tracers were utilized to validate reported (e.g., Fig. 1a–b; **Extended Data Figs. 2, 12, 14a, 25e**). For quantitative analysis, we utilized our in-house informatics tools, *Outspector* ^28,38,42,43,44^, which supports reliable and accurate registration, annotation, and analysis of connectivity data (**Extended Data Figs. 7a, 10**; refer to **Supplementary Methods** for details). This enabled the creation of a comprehensive, accurate, and reliable data, allowing the examination of brain-wide connectivity patterns from different tracer injection locations (refer to **Supplementary Results** for further details).

We also employed the AAV1-Cre-based anterograde transsynaptic tagging method ^74^ to uncover cell-type-specific bi-synaptic pathways. Our findings show that the DPs modulates sympathetic outputs through preautonomic neurons in the PVHd and LHA (DPs→PVHd/LHA→IML) and inhibits vagal parasympathetic outputs via the CEA (DPs→CEA→DMX). Consistent with previous reports ^74^, AAV1-Cre transsynaptic tagging effectively examines unidirectional synaptic connections, suitable for pathways in this study. Combining this method with single-neuron recording and CRACM techniques, we confirmed direct DPd innervation of PVH CRH neurons (DPd→CRH neurons (Fig. 5g) and mapped convergent inputs to DP neurons from cortical regions like AUDv, ENTl, and PIR (Fig. 6a–c). Lastly, we demonstrated DP’s role in regulating CORT release and social interactions (Fig. 5h–i). In summary, our study integrates structural and functional circuit mapping to provide novel insights into the DP’s role within the MPF, highlighting its significance in neural regulation of visceral motor outputs.

### The concept of the primary visceromotor cortex.

**Figure 7:**
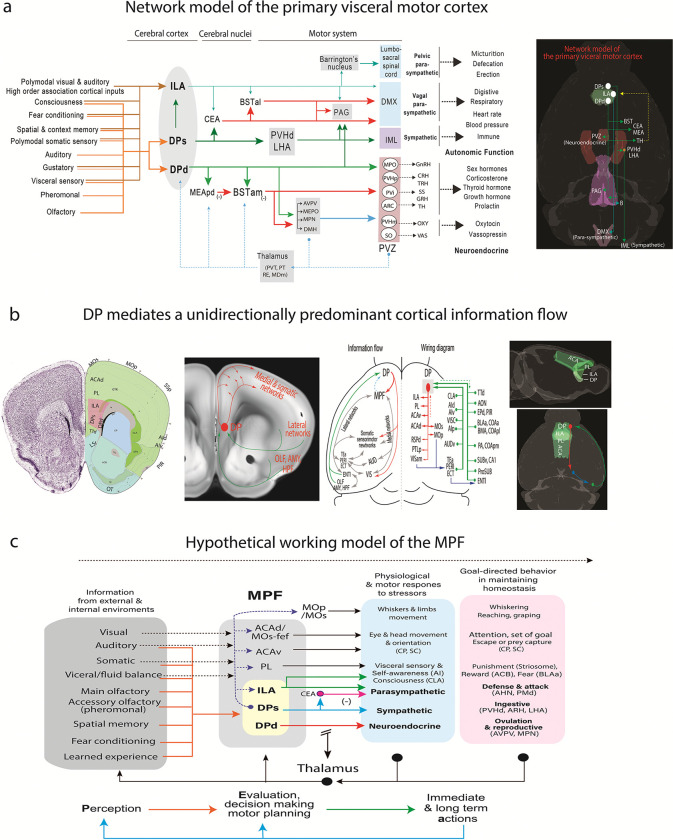
Proposed working model of the MPF based on network analysis. **a,** Schematics showing that the DPs, DPd, and ILA constitute the primary visceral motor cortex, which governs neuroendocrine, sympathetic, and parasympathetic outputs. The right panel depicts the schematic network pathways of the three components of the primary visceral motor cortex within the whole mouse brain. **b,** Left panel shows the anatomic location of the DPs and DPd in 2D. Middle panels show a schematic and wiring diagram that illustrate the DP as a critical junction node, mediating a predominantly unidirectional flow of information from caudal to rostral and lateral to medial within cortico-cortical pathways. The DP bridges communication between lateral cortical subnetworks with both medial cortical and somatic motor cortical subnetworks. Additionally, it facilitates the transfer of information from the olfactory cortex, amygdala, and hippocampus to the MPF. The right panel shows the anatomical locations of the DP, ILA, and other MPF areas in 3D views. **c,** A proposed unitary MPF model based on network analysis. The DP serves as an integrative center, receiving comprehensive information from both external and internal environments. It transmits this information to other cortical areas including ILA, PL, ACAv, ACAd, MOs, and MOp. Each of these MPF areas, along with MOp/MOs, carries out specific physiological and motor responses to various stress stimuli and plays a role in regulating different goal-directed behaviors to ensure long-term homeostasis through their projections to different brain structures (see main text for details). For example, the ACAd (and its adjacent MOs-fef) and ACAv send dense projections to the dorsomedial striatum or caudoputamen (CP in rodent) and superior colliculus (SC), which aid in coordinating eye and head movement during navigation ^42,45,86,92^ and attention ^3^. The ACA and its subcortical targets, such as the SC, are also crucial in animals escaping or prey capturing behavior ^45,86^. These downstream effectors send projections to the thalamus, creating a feedback loop that allows for the regulation of MPF activities. This network model aligns well with the classic Perception-Evaluation-Action (PVA) model ^3^. Abbreviations: ACB, nucleus accumbens; AHN, anterior hypothalamic nucleus; AI, agranular insular cortex; ARH, arcuate nucleus; AVPV, anteroventral periventricular nucleus; B, Barrington’s nucleus; BSTam, al, rh, the anteromedial, anterolateral, rhomboid nuclei of the bed nuclei of the stria terminalis; BLAa, anterior basolateral amygdalar nucleus; CEA, central amygdalar nucleus; CLA, claustrum; CP, caudoputamen; DMH, dorsomedial hypothalamic nucleus; DMX, dorsal motor nucleus of the vagus nerve; MEPO, median preoptic nucleus; MPN, medial preoptic nucleus; MPO, medial preoptic area; IML, intermediolateral column of the spinal cord; LHA, lateral hypothalamic area; PMd, dorsal premammillary nucleus; PVHd, descending part of the hypothalamic paraventricular nucleus; PVHm & PVHp, magnocellular & parvicellular divisions of the hypothalamic paraventricular nucleus; PVi, intermediate part of the hypothalamic periventricular nucleus; PVZ, hypothalamic periventricular zone; SC, superior colliculus; SO, supraoptic nucleus. See Table 1 for additional abbreviations.

The most striking finding of this study is the distinct input/output connectivity pattern of the DP compared to its adjacent TTd, ILA and other MPF areas. Additionally, two newly defined subdivisions, DPs and DPd, also exhibit distinct global connectivity patterns. Based on these findings, we propose the concept of *the primary visceromotor cortex* (Fig. 7a). Although the notion of the visceromotor cortex has been speculated ^4,16^, our comprehensive mapping of the MPF determined its main components and their network organization. We propose that the DPd, DPs, and ILA compose the core components of the primary visceral motor cortex, regulating neuroendocrine, sympathetic, and parasympathetic functions in a synchronized manner, thereby, governing different aspects of visceral motor activities. This network model follows a similar organizational logic to that of the primary motor cortex (MOp) in its control of somatic movements of the head, neck, and limbs ^59^.

Firstly, the DPd, DPs, and ILA generate direct projections to motor neuron pools or premotor neurons controlling neuroendocrine, sympathetic, and parasympathetic activities. For the first time, we demonstrate that one MPF area, the DPd, generates monosynaptic projections to neuroendocrine cells in the PVH, as well as the rest of the hypothalamic neuroendocrine zone (Fig. 7a), enabling direct cortical regulation of neuroendocrine activities, such as releases of corticosterone, growth hormone, sex hormones, oxytocin and vasopressin. Meanwhile, the DPs densely innervates preautonomic neurons in the PVHd and LHA, presumably regulating sympathetic outputs through their feedforward projections to the preganglionic sympathetic motor neurons in the IML (DPs→PVHd/LHA→IML). Simultaneously, the DPs inhibits parasympathetic outputs through bi-synaptic feedforward projections via the CEA (DP→CEA→DMX/NTS). Conversely, the ILA generates excitatory monosynaptic inputs to the DMX (ILA→DMX), thereby driving vagal parasympathetic outputs. Collectively, the DPs and ILA appear to coordinate and regulate autonomic activities associated with the digestive, respiratory, and cardiovascular systems. Notably, the ILA and DPd, but not DPs, also directly innervate Barrington’s nucleus, which governs lumbosacral parasympathetic activities, such as micturition, defecation, and erection ^76,84^.

Secondly, similar to the way MOp regulates motor functions through its projections to the basal ganglia, the DPd, DPs, and ILA also generate dense projections in a topographic manner to several cerebral nuclei (striatal or pallidal-like structures with GABAergic projection neurons ^59^), such as the CEA, MEA, lateral septum (LS), ACB and BST, through which they regulate visceral motor activities, innate behavior, and reward. Among them, the BSTam, which receives dense inputs from the DPd, is well known for its role in the regulating neuroendocrine activities ^59,64,77^. The CEA and BSTal, which receive dense inputs from the DPs, play essential roles in the regulating autonomic activities through their projections to the DMX and other brainstem structures ^59,63^. In addition, the DPd and ILA generate dense projections to the MEA and BSTp (the ILA also densely innervates the LS), which in turn project to the hypothalamic medial behavioral column to regulate defensive behaviors ^48,59,85^. Like direct projections of the MOp to the subthalamic nucleus (STN) and SC, the DPd, DPs, and ILA also project densely to the LHA (in the vicinity of STN) and PAG (in the vicinity of SC), which contain preautonomic neurons involved in regulating cardiovascular, respiratory, and other autonomic functions.

The DPd, DPs, and ILA maintain extensive bidirectional connections with several thalamic nuclei, including the RE, PVT, and PT, which relay hypothalamic and brainstem information related to homeostasis (e.g., visceral sensory input, fluid balance) and social behaviors (e.g., defensive, reproductive) ^59^. The PVT and PT receive dense inputs from the PB and NTS, key regions for processing visceral sensory information. Additionally, the PB exhibits bidirectional connectivity with both the DP and ILA, as shown in this study and in previous work ^56^.

Refer to the **Supplementary Discussion** for additional details on how DPs, DPd, and ILA are organized into highly synchronized neural networks that coordinate three basic classes of social behavior: ingestive, reproductive, and defensive.

### Unified MPF model for controlling motivated behavior.

Our understanding of the MPF, or the overall prefrontal cortex, has been hindered by the lack of a unified working model ^8^. Based on the newly constructed, comprehensive whole-brain wiring diagram, we propose a testable hypothetical network model to understand how different MPF components coordinate and synchronize into a unified system, regulating physiological and motor actions in response to environmental and social challenges.

Firstly, our data reveals that the DP mediates a predominantly unidirectional cortical information flow (Fig. 7b). In particular, the DP receives and integrates extensive inputs from various cortical areas along the lateral cortical mantle, both dorsal and ventral to the rhinal fissure. These areas include the olfactory cortex, ENTl, amygdala, subiculum, and neocortical regions within the lateral cortical subnetworks ^38^, such as PERI, ECT, TEa, AUDv, AIp, AId, and AIv. As a result, the DP processes a wide range of information from both external and internal environments, including olfactory, pheromonal, visceral sensory, auditory, fear conditioning, and spatial orientation signals. Integrating these diverse modalities is crucial for perception, risk evaluation, decision-making, and memory formation ^3^.

In turn, the DP generates dense projections to other MPF and cortical areas, through which to regulate motor outputs (Fig. 7c): (1) The DPd, DPs, and ILA constitute the primary visceral motor cortex, regulating neuroendocrine and autonomic responses as discussed above. (2) The ILA and PL send dense projections to the ACB, which plays a critical role in reward processing through its connections with the VTA^70^. (3) The ACAv and ACAd form key components of the dorsomedial PFC ^8^, which, along with the adjacent MOs-fef as well as other cortical areas within the medial cortical network (e.g., PTLp, RSPv), project robustly to the dorsomedial striatum (which project sequentially to the rest of the basal ganglia ^42^) and SC ^45^. Together, these projections regulate and coordinate eye, head, and neck movements, essential for attention, spatial orientation, and navigation ^3,59^. (4) The DPs, as well as other MPF areas, also provide significant inputs directly to both MOs and MOp, contributing to the control of somatic movements involving the body trunk and limbs. Altogether, these connections provide the necessary network substrates to synchronize visceral and somatic motor actions associated with different goal-directed behaviors ^3,10,86^. Refer to the **Supplementary Discussion** for additional details on how *the purported unified MPF model synchronizes motivated behavior.*

We observed that while the DP mediates ventral-to-dorsal information flow via the olfactory cortex to MPF, at the caudal end of the cerebral cortex, three adjacent areas near the rhinal fissure—TEa, ECT, and PERI—collectively referred to as the rhinal cortex ^49^, mediate dorsal-to-ventral information flow. They receive convergent inputs from sensory cortices such as VIS, AUD, and SSp, and in turn, project to the ENTl ^38^. The ENTl then relays this information to the DP and other MPF areas, thereby facilitating circulation of global cortico-cortical information flow. However, the hypothesis of this predominantly unidirectional cortical information flow requires further validation.

### Related to the primates and human.

According to their connectivity, we suggest that the DPd, DPs, and ILA, as well as the PL, constitute parts of the ventromedial prefrontal cortex (vmPFC) in primates and humans ^4,8,12,16,17,87^, although the vmPFC connectivity has not been examined with equivalent granularity in these species. Subgenual regions of the vmPFC, including Broadman area 25, are identified as components of a central autonomic network responsible for monitoring visceral functions ^8,17,87^; but it remained unclear how vmPFC regulates neuroendocrine activities. Previous investigations into the role of the vmMPF in mood disorders in humans has yielded mixed results ^12,88,89^. Here, our results indicate that the anatomically adjacent DP and ILA, presumably contribute significantly to discrepancies due to their distinct neural circuits controlling neuroendocrine and autonomic functions with opposing actions. ILA activation reduces endocrine and cardiovascular stress responses ^1^, while DP activation increases plasma corticosterone levels as shown in the present study and in a previous report ^21^.

Recent studies in rodents suggested that the DP and TTd are activated by social interactions and various stressors like acute restraint stress, looming sounds, fear memory, predatory threats, and opioid addiction ^19,20,21,22,23,24^. Our study also showed that DP neurons are activated by both social (sex dependent) and non-social stimuli. In contrast, the ILA does not show sex-dependent social responses. Nevertheless, it is necessary to determine the homologous parts of the DPs, DPd, ILA, and other MPF areas in primates and humans using modern technologies ^8^ and to validate their functional roles in proper physiological and social responses to environmental stimuli in maintaining homeostasis and navigating complex social landscapes ^3,48,90^. As we show, the DP sits in a critical node bridging global cortical information flow (Fig. 7b). If the DP, along with the ILA (or vmMPF in humans and primates), is affected by lesions, it can disrupt the integration of sensory information and perception of both external and internal stimuli. As a result, their connections with other components of the MPF and cortical areas may also be disrupted. This disconnection between perception, evaluation, and action can lead to excessive, inappropriate, or insufficient emotional and behavioral responses (Fig. 7c, also see Supplementary Discussion). This is often observed in various psychiatric disorders, including classic Phineas Gage-like personality disorders, major depressive disorder (MDD), as well as PTSD ^3,5,13^.

## Methods

### Animals

Animals used in this study are C57BL/6J male and female mice at ages of 2–4 months (Jackson Laboratories). Animals were housed in a temperature (21–22 °C), humidity (51%), and light (12-hour light/dark cycle: lights on at 6:00 am/off at 6:00 pm) controlled room. Food and water were given ad libitum. Upon arrival at the facility, animals were allowed a minimum of 1 week to adapt to the housing environment before surgeries were performed. All experiments were conducted according to the standards set by the National Institutes of Health Guide for the Care and Use of Laboratory Animals and the institutional guidelines of the University of Southern California (USC) and University of California Los Angeles (UCLA).

MORF3 (C57BL/6-Gt(ROSA)26Sortm3(CAG-sfGFP*)Xwy/J), generated by Dr. X. William Yang's lab at UCLA ^1^, is a Cre reporter mouse line that utilizes a mononucleotide repeat frameshift (MORF) as a translation switch for cell labeling *in vivo*. MORF3 mice express a Cre-dependent tandem "spaghetti monster" fluorescent protein with 20 V5 epitopes (smFP-V5) preceded by a polycytosine repeat (C22) MORF switch under the control of a CAG promoter. Cre recombination combined with a spontaneous frameshift results in sparse and stochastic labeling of neural cells.

Two other transgenic mouse lines, including Vglut2-Cre (B6J.129S6(FVB)-Slc17a6tm2(cre)Lowl/MwarJ) and Ai14 B6.Cg-Gt(ROSA)26Sortm14(CAG-tdTomato)Hze/J, were purchased from JAX. The breeding colonies of these mouse lines were established at UCLA.

### Tracers

Phaseolus vulgaris leucoagglutinin (PHAL; 2.5%; Vector Laboratories) was used as the main chemical anterograde tracer. Chemical retrograde tracers included cholera toxin subunit b (CTB AlexaFluor 647 conjugate, 0.25%; Invitrogen) and Fluorogold (FG; 1%; Fluorochrome, LLC). Each anterograde and retrograde tracer has distinct characteristics and exhibits varied neurotropism that can meaningfully affect connectivity results. For example, anterograde AAVs label fibers of passage, while PHAL does not. These differences underscore the importance of data validation. In this work, the connectivity data were validated in multiple ways to ensure the reliability of the results. Retrograde tracers were placed in regions of anterograde terminations, while anterograde tracers were placed in regions of retrogradely labeled cells. For example, Extended Data Fig. 12 shows retrograde tracer injection in the DPs and DPd that reveal their distinct brain-wide inputs (Brain-wide ROIs→DPs or DPd). To validate these results, Extended Data Fig. 14 shows anterograde tracer injections delivered to those brain-wide ROIs to validate their projections to either DPs or DPd. Further, we have previously demonstrated that PHAL and AAV produce similar brainwide anterograde labeling patterns (see Hintiryan et al., 2021 ^2^ Supplementary Fig. 6).

AAVretro-hSyn-Cre-WPRE (AAV retro Cre; 1.6 × 1013 GC/ml; Addgene #105553), AAV1-Syn-Flex-GCaMP6f-WPRE-SV40, AAV1-hSyn-SIO-stGtACR2-FusionRed, and AAV-Ef1a-mCherry-IRES-Cre (retrograde) ^3^ were produced by Addgene (Watertown, MA, USA).

Viral anterograde tracers AAV encoding enhanced green fluorescent protein (AAV-GFP; AAV2/1.hSynapsin.EGFP.WPRE.bGH) and tdTomato (AAV1.CAG.tdtomato.WPRE.SV40), and AAV1-hSyn-Cre-WPRE-hGh (anterograde trans-synaptic) were packaged by UPenn Vector Core (Philadelphia, PA, USA). AAV5-Ef1a-DIO-hChR2(H134R)-eYFP, AAV9-CAG-FLEX-GFP (Cre-dependent channelrhodopsin expressing AAV) was produced by the University of North Carolina vector core facility (Chapel Hill, North Carolina, USA):

The viruses, which are components of the rabies viral tracing system, were produced by Wickersham Lab at MIT and included AAV2/9-CAG-FlexmKate-T2A-TVA, AAV2/9-CAG-Flex-mKate-T2A-N2c-G, and Rbv-CVS-N2c-DG-GFP (the modified rabies virus). All viral vectors were aliquoted and stored at 80 °C until use. Importantly, rabies virus has been widely used as a tool for reliably mapping presynaptic inputs to a specific brain region or starter cell population ^4,5,6^. While its tropism appears broad, it remains possible that specific cell types in different pathways may be over-or under-represented relative to the performance of other retrograde tracers and viruses (e.g., AAVretro, CAV2, retrobeads, etc) ^7,8^. Moreover, the potential for cytotoxicity within the starter cell population may provide an opportunity for rabies to non-specifically spread to nearby synapses rather than through those specifically making contact with starter cells. To control for these possibilities, retrograde tracing results must be validated using complementary anterograde approaches, such as by tracing axonal connections from the upstream region, or by optogenetically stimulating upstream axons and recording synaptic responses in the target cell population. As such, the data we present were validated in several different ways.

### Stereotaxic surgeries

Surgical and microscopic imaging procedures have been described previously ^9,10,11^. Stereotaxic coordinates of targeted injection centers were determined through the Allen Reference Atlas ^12^ and empirically adjusted when needed. On the day of the experiment, mice were deeply anesthetized and mounted into a Kopf stereotaxic apparatus where they were maintained under isoflurane gas anesthesia (Datex-Ohmeda vaporizer). Prior to the surgery, mice were given one subcutaneous injection of Ketoprofen (4 mg/kg) and a protective ophthalmic ointment was applied to their eyes. For single anterograde tracer injection experiments (PHAL or AAV only) tracers were iontophoretically delivered via glass micropipettes (inner tip diameter 24–32 μm) using alternating 7 sec on/off pulsed positive electrical current (Stoelting Co. current source) for 10 min, and AAVs were delivered via the same method for 2 min (inner tip diameter 8–12 μm). For anterograde/retrograde coinjection experiments (PHAL/CTB-647 and AAV/FG), tracer cocktails iontophoretic injections were made via glass micropipettes (inner tip diameter 28–32 μm) for 10 min (PHAL/CTB-647) or 5 min (AAV/FG).

For quadruple retrograde tracing experiments, at each injection site, 50 nL of the retrograde tracer was individually pressure-injected via glass micropipettes at a rate of 10 nL/min (Drummond Nanoject III). All injections were placed in the right hemisphere. Following injections, incisions were sutured, and mice were returned to their home cages for recovery.

### TRIO tracing (TVA receptor mediated rabies tracing)

To reveal mono-synaptic inputs to projection defined neuronal populations in the dorsal peduncular cortex (DP) (e.g., DP neurons projecting to anterior cingulate cortex, ACA), we used a modified TRIO (tracing the relationship between input and output) strategy ^13^. In brief, AAVretro-Cre was injected into a downstream projection target of DP (e.g., ACA), and Cre-dependent TVA-and RG-expressing helper virus (AAV8-hSyn-FLEX-TVA-P2A-GFP-2A-oG) and mCherry-expressing G-deleted rabies virus (produced by Laboratory of Ian Wickersham at MIT) were injected into the DP to label the DP projection neurons population (1^st^-order) and their brain-wide monosynaptic inputs (2^nd^-order).

### AAV1-Cre-based anterograde transsynaptic tracing

This technique leverages the fact that when AAV1 is injected at sufficiently high concentration into a neuronal population, viral particles will travel down the axons and be released from the synaptic terminals where they can infect postsynaptic neurons. Detailed methodology is as described ^14^. In brief, anesthetized mice were iontophoretically injected with Cre-dependent AAV-FLEX-RFP or GFP in the target structures (e.g., lateral hypothalamic area, LHA), and pressure injected (20–80 nl) with AAV1-Cre in an upstream structure, (e.g., the DP). The AAV1-Cre is transported anterogradely down the axons and is released from the terminals, where it transfects postsynaptic cells that have been infected with high concentrations of Cre-dependent AAV-FLEX-RFP. The scant Cre expression is sufficient to unlock strong fluorophore expression in the downstream neurons, therefore, revealing their axonal projections and terminals. After a three-week post-operative recovery, animals were pentobarbital-anesthetized and perfused. The Cre injection site was verified by staining with mouse anti-Cre recombinase monoclonal primary antibody (Millipore Sigma, MAB3120) and donkey anti-mouse AlexaFluor 647 secondary antibody (Jackson ImmunoResearch, 715–605-150). One caveat to using AAV1-Cre is that the virus can also travel retrogradely and should be used in situations where the connection is known to be primarily unidirectional. In all the experiments in which AAV1-Cre was used in the current paper, the connections were shown to be predominantly unidirectional (BLA/SUBv/AUDv→DP and DP→CEA/LHA/PAG). As an example, we injected AAV1-Cre in the DP and a Cre-dependent AAV in the LHA to show that LHA neurons that receive input from the DP project to the spinal cord (DP→LHA→spinal cord). If LHA projected back to the DP, we possibly would be showing that LHA neurons that project to DP also project to the spinal cord. However, we know that the DP→LHA connection is unidirectional (**Extended Data Fig. 22a**), which lends reasonable confidence to our conclusion that DP→LHA→spinal cord.

See Supplementary Material subsection, *Injection Site Analysis*, for details regarding how injection site locations were accurately determined.

### Tissue preparation and immunohistochemistry

Animals were sacrificed with an overdose injection of sodium pentobarbital (6 mg/kg) 7 days (chemical tracers) or 14 days (viral tracers) following surgeries. Each animal was transcardially perfused with approximately 50 ml of 0.9% NaCl followed by 50 ml of 4% paraformaldehyde solution (PFA; pH 9.5). The brains were post-fixed in 4% PFA for 24–48 hours at 4 °C, after which they were embedded in 3% Type I-B agarose (Sigma-Aldrich) prior to sectioning. Four series of coronal sections were sliced at 50 μm thickness with a Compresstome (VF-700, Precisionary Instruments, Greenville, NC) and prepared for processing. One of the four section series was immunostained for imaging. The series that contained coronal level 53 of the Allen Reference Atlas was selected to maintain similar section level distributions across experiments.

The following primary antibodies were used across the studies: rabbit anti-PHAL (Vector Laboratories, AS-2300, rabbit anti-fluorogold antibody (Millipore Sigma, AB153-I), mouse anti-Cre recombinase antibody Clone 2D8 (Millipore Sigma, MAB3120), rat monoclonal c-Fos antibody (Synaptic Systems, 226 017), mouse anti-Oxytocin antibody Clone 4G11 (Millipore Sigma, MAB5296), rabbit anti-vasopressin antibody (Millipore Sigma, AB1565), and streptavidin for biocytin labeled recorded neurons (Thermo Fisher, S21374)

Briefly, sections were transferred to a blocking solution containing normal donkey serum (Vector Laboratories) and Triton X-100 (VWR) for 1 hour. Following three 5-minute rinses, sections were incubated in a KPBS solution comprised of donkey serum, Triton, and a primary antibody at proper solution (e.g., 1:1000 concentration of rabbit anti-PHAL antibody) for 48–72 hours at 4 °C (Vector Laboratories, AS-2300). Sections were rinsed three times in KPBS and then soaked for 3 hours in the secondary antibody solution, which contained donkey serum, Triton, and a corresponding secondary antibody solution with proper solution (e.g., 1:500 concentration of anti-rabbit IgG) conjugated with AlexaFluor 488, 555 or 647 (Invitrogen, 488: A-21206, 647: A-31573). Sections were again rinsed with KPBS three times. All selected section series were counterstained with a fluorescent Nissl stain, NeuroTrace 435/455 (NT; 1:500; Invitrogen, N21479). The sections were then mounted and coverslipped using 65% glycerol.

### RNAscope experiments

Deeply anesthetized animals were perfused, and brains were post-fixed in 4% PFA overnight followed by sequential immersion in 15% and 30% sucrose for cryoprotection. Brains were then sectioned (20 μm) and RNAscope Multiplex Fluorescent v2 assay was performed following the manual provided by Advanced Cell Diagnostics. In brief, slides containing tissue sections were post-fixed in the pre-chilled 4% PFA for 15 min at 4 °C, and then dehydrated in a series of ethanol solutions 50%, 70% and 100% for 5 min at RT. Hydrogen Peroxide was applied to each section for 10 min at RT, and target retrieval was then performed at 99 °C for 5 min. Protease III was then added to the samples and incubated in a HybEZ oven for 30 min at 40 °C. The subsequent RNAscope assay was composed by multiple steps of hybridization (probe and AMP binding) and signal development (HRP channel binding), each followed by washing the slides in 1× wash buffer as described in the manufacturer protocol (Multiplex fluorescent v2 kit). Opal fluorophores (Akoya Biosciences) were diluted at 1:1500 to visualize RNA signals. Following RNAscope assay, sections were counterstained with DAPI and mounted. Images were acquired using a Dragonfly High Speed Confocal Microscope System (Andor). The following probes were used in this study: Cux2, Etv1, Tle4, SS, CRH, TRH.

### Image acquisition and 2D processing

All 50 μm sections were scanned as high-resolution virtual slide image (VSI) files using an Olympus VS120 high-throughput microscope. Post-acquisition processing methods, including image registration, tracer segmentation, and quantification, are described in detail below. Briefly, all images were flipped to ensure that the injection site and its corresponding ipsilateral labeling were in the correct right hemisphere. This was completed by the VS-Desktop software that accompanies the microscope. Images were then imported into *Outspector–*our in-house software for image processing (see Supplementary Methods subsection *Outspector: our proprietary 2D image processing pipeline*) and processed on a high-performance computing cluster. A first pass fully automatic registration was executed and subsequently its results were inspected and fine-tuned when needed. Tracer segmentation was performed on registered images with default parameters. Segmentation results were also inspected, followed by interactive parameter adjustments if needed. Segmented pixel counts and cell counts in each brain region at all section levels were generated and used for analysis.

### Outspector

*Outspector* is our informatics workflow specifically designed to reliably warp, reconstruct, annotate, and analyze the labeled pathways in a high-throughput fashion. Tissue sections from each analyzed case were assigned and registered to standard templates of the corresponding ARA levels. All images shown in this manuscript are from the raw data of unwarped, unregistered VSI images. Threshold parameters were individually adjusted for each case and tracer. Adobe Photoshop (RRID:SCR_014199) was used to correct conspicuous artifacts in the threshold output files. Each color channel was brightness/contrast adjusted to maximize labeling visibility (Nissl Neurotrace 435/455 is converted to brightfield), and TIFF images are then converted to JPEG file format. The detailed calculations used for all *Outspector* processing steps and analysis can be found in the Supplementary Methods subsection *Outspector: our proprietary 2D image processing pipeline*.

### Quantifying colocalization

Using spots function on Imaris, thresholds were set to select the majority of positive cells for each channel (405, 488, 561, and 642). After automatic detection, manual quality control was necessary. The spatial relationship between DAPI in 405 and RNA puncta for Fezf2 was used as a visual aid to correct false negatives and remove false positive spots from the automatic detection. Each channel was quality controlled multiple times to ensure accuracy.

### Tissue processing and image for 3D single neuron labeling

To elucidate the finely detailed morphology of DP neurons we injected either AAV-retro Cre or AAV1-Cre in MORF3 mice. Three to four weeks following injections animals were perfused. We also acquired two neurons derived from CamK2/MORF3 double transgenic mice (provided by the Yang lab at UCLA). In this model, between 1% and 5% of the excitatory neurons in the cerebral cortex are labeled with MORF3. Immunostaining with the V5 antibody allows the visualization of the finely detailed neuronal morphology of these MORF3 neurons. This approach allowed us to image, reconstruct, and analyze the cell-type-specific morphological features of DP cortical projection neurons. Following the same strategy, we also revealed sparsely labeled neurons with finely detailed dendritic morphology in the lateral hypothalamic area.

The SHIELD clearing protocol was used for the 3D tissue processing ^15^. Mice were transcardially perfused with cold saline and SHIELD perfusion solution. Brains were dissected, subjected to a SHIELD-based brain clearing method, and immunostained for V5 (to reveal MORF3 with secondary antibody conjugated with Alexa Fluor 647) via eFLASH ^1,15,16^. Brains were processed using Smartclear and SmartLabel machines (LifeCavas SmartBatch). Whole brains were imaged with a Life Canvas SmartSPIM lightsheet microscope at 15x (0.42μm × 0.42μm × 0.42 μm). Three lasers (488, 555, 647 nm) were used, respectively, to image Syp-EGFP axonal terminals, tdTomato-positive cortical neurons and their axons, and MORF3-positive postsynaptic neurons.

For acquiring images on thick sections, the whole brain was cut into 500 μm sections and were cleared in the SDS buffer at 37°C for 72 hours. The sections were then washed three times with KPBS and incubated in KPBS at 4°C for 24 hours. Sections were mounted and cover-slipped onto 25×75×1mm glass slides with an index matching solution 100% (EasyIndex, LifeCanvas Technologies, #EI-Z1001). Sections were imaged with a high-speed spinning disk confocal microscope (Andor Dragonfly 202 Imaging System, Andor an Oxford Instruments Company, CR-DFLY-202–2540). 10x magnification (NA 0.40, Olympus, UPLXAPO10X) was used to acquire an overview after which 30x magnification (Olympus, UPLSAPO30xSIR, NA 1.05, W.D.: 0.80 mm) at 1μm z steps or 60x magnification (Olympus UPLASAPO60XS2, NA: 1.30, W.D.: 0.30 mm) were used to acquire high resolution images for fine detailed dendritic morphology and putative synaptic connections. Sections were imaged with four excitation wavelengths (nm): 405 (blue Nissl background), 488 (for AAV-GFP), and 647 (far red for MORF3) with respective emission detection wavelengths of 450, 525, and 600.

### 3D reconstructions, visualizations, and analysis of neuronal morphology

Manual reconstruction of neurons was performed using Vaa3D ^17, 18^ or Fast Neurite Tracer ^19^, followed by morphological repairing, sorting and typing of the dendritic arbors. Reconstructions were finalized by loading them in Imaris and neuTube ^20^ and finalized into the widely used swc file format ^21^. In total, 48 reconstructed DP neurons were morphologically analyzed in MATLAB, leveraging functions from the Trees Toolbox environment ^22^. Specifically, new analysis scripts measured all neurons across a series of parameters including total dendritic wiring length, number of branches, average branch angle, average root angle, branch asymmetry, average branch order, average Strahler order, distance from midline. Then the coefficients of variances across the parameters were measured, followed by classification using K-means clustering.The size and complexity of dendritic arbors, defined by total dendritic length and the total number of branches, emerged as the most varied. We then classified all the neurons, first based on dendritic arbor size and complexity, followed by *Superficial* vs *Deep* layer classification based on distance of all cell-bodies from the midline. Both classifications created two neuronal clusters each. *Superficial* vs *Deep* layer neurons were then compared for size, complexity and dendritic orientation using nonparametric Wilcoxon Signed Rank test. The p values from the test were corrected using the False Discovery Rate (FDR) correction to account for multiple comparisons. Similarly, the two morphological clusters (small-simple and large-complex) were compared for their distance from midline.

### In vitro patch-clamp slice recordings

In wildtype animals, AAV-hSyn-ChR2-YFP (UPenn Vector Core) was injected into the lateral entorhinal cortex (ENTl), while AAV1-Cre (anterograde transsynaptic Cre) was injected into either the ventral auditory cortex (AUDv) or piriform cortex (PIR) of different animals. In separate experiments, the AAV-hSyn-ChR2-YFP was injected into the DP of CRH-Cre Ai14 mice.

Two weeks following channelrhodopsin and tracer injections, acute brain slices were prepared for electrophysiological recordings. Following anesthesia with isoflurane, the animal was decapitated, and the brain was rapidly removed and immersed in ice-cold high sucrose slice solution (in mM): 208 sucrose, 2.5 KCl, 1.25 NaH_2_PO_4_, 26 NaHCO_3_, 1.3 MgCl_2_, 8 MgSO_4_, and 10 glucose; saturated with 95% O_2_ and 5% CO_2_; pH = 7.4. Brain slices (350 μm thickness) were cut in the coronal plane using a vibrating microtome (Leica VT1000S). Slices were allowed to recover for at least 30 min in a submersion chamber filled with warmed (32–34 °C) ACSF containing (in mM) 130 NaCl, 3 KCl, 1.25 NaH_2_PO_4_, 26 NaHCO_3_, 2 CaCl_2_, 2 MgCl_2_ and 10 glucose, oxygenated with 95% O_2_ and 5% CO_2_, pH 7.2–7.4, 290–310 mOsm, and then cooled gradually to room temperature until recording. The presence of RFP and ChR2 (YFP) labelling was determined respectively with green and blue fluorescence excitation in the slices before recording. Patch pipettes with ~3–4 MΩ impedance were used for whole-cell patch clamp recordings. Glass pipettes contained a Cs-methanesulfonate-based internal solution with the following salt concentrations (in mM): 130 Cs-methanesulfonate, 10 CsCl, 4 NaCl, 1 MgCl_2_, 5 150 MgATP, 5 EGTA, 10 HEPES, 5 GTP, 10 phosphocreatine, and 0.1 leupeptin (pH 7.2 with 151 CsOH, 270 mOsm)) and biocytin (0.2%). For isolation and recording of monosynaptic responses to blue light stimulation tetrodotoxin (Na^+^ channel blocker, 1 μM) and 4-aminopyridine (K^+^ channel blocker, 100 μM) were added to the external solution. Signals were recorded from red-labelled neurons with a MultiClamp 700B amplifier (Molecular Devices) running pClamp software (version 10.5) under voltage clamp mode at a holding voltage of −70 mV for excitatory currents, filtered at 1 kHz and sampled at 10 kHz. Blue light (470 nm) stimulation was delivered using a 0.5 ms pulse, at ~1 mW power, for 5 trials, delivered via a LED (CoolLED). Signals were analyzed using Clampfit (version 10.5). For responding neurons, peak responses were averaged across trials. To demonstrate that light-evoked synaptic responses were indeed excitatory, selective glutamate receptor antagonists CNQX (20 μM) for AMPA receptors and APV (50 μM) for NMDA receptors, were added to the bathing solution.

After recording, the slices were fixed with PFA and then transferred to PBS for biocytin staining to reveal and validate the location of the recorded neurons. The biocytin staining also revealed the morphology of the recorded DP or PVH neurons.

### Optogenetic stimulation and measurements of plasma corticosterone hormone

A total of 15 mice (male C57Bl/6, 2-month-old) were randomly assigned to either the Experimental (EXP) or Control (CON) group. Each of the animals received bilateral injections of AAV1-Cre into the ventral subiculum (SUBv) and the anterior basolateral amygdalar nucleus (BLAa), allowing Cre to anterogradely transport to and tanssynaptically label DP neurons. The animals also received a bilateral injection of Cre-dependent AAV-FLEX-ChR2 (30 nl) into the DP. Consequently, only DP neurons postsynaptic to the SUBv and BLAa express ChR2 and generate projections to the hypothalamic neuroendocrine zone, including the PVH. Optic fibers (200 μm core diameter, 0.37 numerical aperture; Inper) were implanted above the injection site at a depth of −1.6 mm ventral to the bregma skull surface and secured to the skull using super glue and dental cement.

The optogenetic stimulation was performed 7–8 weeks after the surgery. Prior to testing, the mice were individually habituated to the testing cage (new mouse cage) and optic fibers over four consecutive days, for 30 minutes daily. On test day, the mice were habituated to the testing cage alone for 2 hours and attached to optic fibers for 15 min before optogenetic stimulation. A 473-nm laser, pulsing at 20Hz with 15-ms pulses, was applied for 30s ON and 30s OFF cycles, for a total of 20 min. The light irradiance was around 4–5 mW mm^−2^ in the target region. Following laser stimulation, the optic fibers were removed, and the mice remained undisturbed in the testing cage for 25 min before sacrifice. To minimize circadian rhythm effects on corticosterone levels, animals were tested between 1.5 to 5 hours after lights turned off in the dark phase, with control and ChR2 groups counterbalanced during the test.

### Serum collection and corticosterone (CORT) assaying

Following rapid cervical dislocation, terminal trunk blood was collected with heparinized microcapillary tubes (Fisher Scientific) and transferred into microfuge tubes, which were left on ice for at least 15–20 mins. The blood samples were centrifuged at 2,500 rpm for 15 min at 4 °C. The supernatant (serum) was collected and transferred into new tubes and the serum samples were frozen at −80 °C. The samples were packed and shipped on dry ice to the Center for Research in Reproduction Ligand Assay and Analysis Core at the University of Virginia School of Medicine, where they were processed (in duplicate) utilizing a radioimmunoassay kit for corticosterone measurements.

### Social interaction experiments

#### Surgery.

Male (VGLUT2-Cre, 6–8-week old) were anesthetized using a 1–2% isoflurane-oxygen mixture and injected unilaterally with 350 nl (1 nl/sec) pAAV.Syn.Flex.GCaMP6f.WPRE.SV40 (Addgene: 100833-AAV1) into the dorsal peduncular cortex (DP). Thirty minutes post-virus injection, a 6.1mm long (0.5mm diameter) relay lens (Inscopix) was implanted over the DP: AP +2.1mm, ML 0.6mm, DV-3.2mm. The lens was securely affixed to the skull using cyanoacrylate glue and dental cement, and then covered with Kwik-Sil (WPI).

Three weeks later, animals were anesthetized as described previously and a UCLA V4 miniscope with an attached baseplate was positioned over the lens to visualize GCaMP6f expression. The focal plane was adjusted to optimize cell visibility, and the baseplate was secured in place with dental cement. A plastic cap was used to cover and protect the lens over the baseplate.

#### Behavioral tests.

Mice with lens implantation in DP were habituated to handling and miniscope for 4–5 days. On the day of the open field test, the miniscope was attached to the baseplate and mice freely ran in an open arena (45cm × 45cm × 30cm) for 20 mins. On the day of same sex social interaction, mice performed social interaction counterbalanced with object exploration in the same arena as the open field test for 8 mins per session. The next day, mice performed social interaction with opposite sex social targets for 8 mins. Each social trial included an 8-minute recording session, beginning with a 1-minute period of the subject mouse alone, followed by a 7-minute interaction with the social partner. On the day of same sex social interaction counterbalanced with object exploration, after the first trial, the subject mouse rested with the LED off for approximately 5 minutes before starting the second trial. Calcium imaging and behavior (miniCam) were recorded simultaneously at 30 Hz.

#### Pre-processing and extraction of calcium signals.

Calcium imaging videos were motion corrected (NoRMCorre: https://github.com/flatironinstitute/NoRMCorre ; MiniAn (https://github.com/denisecailab/minian ) and analyzed with extended constrained non-negative matrix factorization (CNMF-E) algorithm based packages (MiniscopeAnalysis: (https://github.com/etterguillaume/MiniscopeAnalysis ; MiniAn (https://github.com/denisecailab/minian ) to identify cells and extract cell activity. Denoised and demixed trace data were used for further analysis.

#### Identification of behavior responsive cell.

To determine whether an individual neuron’s activity was significantly modulated by social interaction, we used receiver operating characteristic (ROC) analysis to calculate an area under curve (AUC) value for denoised calcium trace correspondence to binarized manually scored interaction bouts. The interaction bouts were defined when the imaged mouse actively investigated the social target/object within a distance of ¼~⅓ of mouse body length. To determine whether the AUC values indicated that the neuron was significantly excited or inhibited by the interaction, we compared observed AUC values to a null distribution derived from circularly shuffled calcium signals (1000 shuffles). Significance was determined using percentile thresholds, classifying neurons as significantly responsive if their auROC values surpassed the 97.5th percentile for excited neurons or 2.5 percentile for inhibited neurons. Neurons that were neither excited nor inhibited were deemed non-responsive.

#### Calcium event calculation.

Calcium transients were identified using automatic detection algorithms in Matlab (peakfinder) (https://github.com/GradinaruLab/striatum2P ).

### DP neuronal activities in response to social and environmental stimuli

#### Male mice in response to social stimuli.

Twenty-four male mice, aged between 12 and 14 weeks, were used for the study. To reduce potential stress by the testing environment, each mouse was habituated to a clean cage for four consecutive days, spending 2 to 4 hours in the cage and with approximately 30 minutes in the recording position daily. On the testing day, the mice were habituated in the clean cage for about 2 hours prior to the experiment. Unfamiliar male mice, female mice, and P7 pups served as the social stimuli. Each mouse was exposed to three instances of the same type of social stimulus, lasting about 6 minutes each, for a total duration of 20 minutes, with six mice in each group. In the control group, the cages were open three times as well, but without introducing any intruders. The mice remained undisturbed in their individual cages for 80 minutes before perfusion.

#### Acute Restraint Stress.

Animals (n=4, male) subjected to acute restraint stress were removed from the colony room, transferred to a behavioral testing room for one hour, and then placed in a Tailveiner^®^ restrainer for mice (Braintree Scientific). This device consists of a clear tube, within which a plug was adjusted to limit voluntary movement. Acute restraint stress was applied for 30 minutes and terminated 30 minutes before the animals were sacrificed. Throughout all studies, control mice (n=4, male) underwent numerous sensory manipulations that were carried out in parallel with those for the mice in the restraint stress group.

### Acute looming sound stimuli in comparison with white noise and foot shock

#### Subjects.

TRAP2-CreER ^23^ and Ai14 mice were acquired from the Jackson Laboratory (www.jax.org/strain/030323; www.jax.org/strain/007908). Homozygous TRAP2-CreER and Ai14 mice were mating to generate TRAP2-CreER:Ai14 offspring.

#### Drug Preparation.

4-hydroxytamoxifen (4-OHT; Sigma, Cat# H6278) was dissolved at 20 mg/mL in ethanol by shaking at 37°C for 15 min, then corn oil was added to give a final concentration of 10 mg/mL, and the ethanol was evaporated by vacuum under centrifugation. 4-OHT (intraperitoneally (i.p.). 50 mg/kg) was injected in all TRAP2-CreER experiments ^23,24^.

#### Stimulation for TRAP.

All the mice automatically divided into 2 groups: Control and Foot shock-based fear conditioning. For auditory stimulation (Noise group), on day1 and 2, the TRAP2:Ai14 mice were adapted to custom-made boxes in a sound-proofed room for one hour per day. On day3, the mice were placed in their home cage for 12 hours in a sound-proofed room before white noise exposure. The mice were then placed in the custom-made boxes and exposed to one-hour broadband white noise stimulation (70 dB SPL).

For the foot shock group, on day1, mice were acclimated in custom-made boxes in a sound-proofed room for one hour. On day2 (conditioning), the mice were put inside custom-built boxes with a metal grid floor. After 10 min habituation to the conditioning chamber, the animals were exposed to the 20 s noise (70 dB SPL) co-terminated with a 0.75-mA foot shock (5Hz for 1 s with the duration of each pulse = 100 ms) for 5 times ^25^. On day3, the mice were only subjected to one-hour broadband white noise exposure. Thereafter, mice received an injection of 4-OHT (i.p., 50 mg/kg) and returned to their home cage. The cage was moved back to our animal facility 12 hours after injection of 4-OHT. Sound stimulation and injection of 4-OHT were conducted in a sound-proofed room. On day12, the mice were further presented with looming sound stimuli. A train of ten consecutive crescendo sound stimuli (with intensity increased linearly from 20 to 70 dB SPL in 0.4 s and followed by 0.6 s interval with intensity at 20 dB SPL) was repeated for ten minutes with an interval of 10-s between the train stimuli. The animal was sacrificed after a 1.5-hour rest.

Control mice were treated the same procedures as the auditory stimulation group but without any sound stimuli.
